# Precise Control of Drug Release in Machine Learning‐Designed Antibody‐Eluting Implants for Postoperative Scarring Inhibition in Glaucoma

**DOI:** 10.1002/adhm.202502689

**Published:** 2026-01-21

**Authors:** Mengqi Qin, Wenbing Jiang, Kai Xin Thong, Zeynep Ulker, Brihitejas Patel, Cynthia Yu‐Wai‐Man

**Affiliations:** ^1^ Faculty of Life Sciences and Medicine King's College London London UK

**Keywords:** drug delivery, glaucoma filtration surgery, machine learning, monoclonal antibodies, ophthalmology, pharmacology, polymers

## Abstract

We have developed a scalable, smart subconjunctival micro‐cylindrical implant system composed of polycaprolactone (PCL) and polyethylene glycol (PEG) for sustained delivery of basic fibroblast growth factor monoclonal antibody (FGFb mAb), a promising anti‐fibrotic agent. This delivery platform allows precise prediction of drug release profiles through machine learning analysis based on key fabrication parameters, such as polymer composition, implant dimension, and drug content. Among the tested machine learning algorithms, LightGBM outperforms others in predicting drug release kinetics (R^2^ = 0.9000 ± 0.0058). Besides, this model effectively elucidates the combined roles of various implant parameters in controlling release behavior and provides insights to guide formulation selection to maximize drug release. The optimized PCL‐PEG implant, combined with 0.1% w/v poly (lactic‐co‐glycolic acid) (PLGA) bio‐coating, exhibits sustained antibody release through synergistic diffusion‐degradation kinetics. In vitro evaluation demonstrates the PCL‐PEG/FGFb mAb@PLGA implant's ability to effectively inhibit 3D‐fibroblast‐mediated collagen contraction and fibrotic genes. In vivo studies in a rat GFS model, along with histological analysis, further validate the implant's efficacy to reduce fibrosis markers, highlighting the implant's potential to modulate wound healing and to prevent postoperative fibrosis. Finally, the PCL‐PEG/FGFb mAb@PLGA implant demonstrates no significant toxicity and good biocompatibility both in vitro and in vivo.

## Graphical abstract

We developed a micro‐sized, biocompatible implant for postoperative sustained delivery of anti‐fibrotic antibodies in glaucoma surgery. Machine learning‐guided optimization of polymer composition, implant geometry, and porosity enabled precise control of drug release. The implant effectively inhibited fibrosis in vitro and in vivo, while showing no toxicity, offering a scalable and minimally invasive strategy to prevent postoperative scarring.

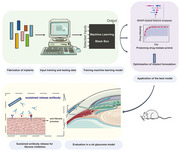

## 1 Introduction

Glaucoma filtration surgery (GFS) remains a cornerstone treatment to lower the intraocular pressure (IOP) in medically uncontrolled glaucoma, yet 46.9% of patients fail surgery after 5 years of follow‐up due to postoperative fibrosis at the conjunctival wound site [1]. The current anti‐fibrotic agents, mitomycin C (MMC) and 5‐fluorouracil (5‐FU), are effective, but carry potential risks of severe complications, such as corneal toxicity, endophthalmitis, and hypotony, primarily due to their nonspecific cytotoxicity [2, 3, 4, 5]. Monoclonal antibodies (mAbs) offer a promising alternative with their high target specificity and minimal systemic toxicity compared to traditional antifibrotic drugs [6, 7, 8]. However, the clinical translation of mAb‐based therapies for GFS is hampered by its rapid clearance, necessitating frequent subconjunctival injections to maintain therapeutic efficacy [9, 10]. To address this challenge, the development of a mAb sustained‐release system is essential to ensure a prolonged local therapeutic effect while reducing the need for repeated injections.

Among ocular drug sustained‐release strategies, such as contact lenses [11], periocular rings [12], and intraocularly administered microspheres [13, 14], millimeter‐sized implants [15, 16, 17, 18] have proven to be well‐suited for clinical ocular applications, providing localized and sustained drug release to target tissues with high drug bioavailability. A critical aspect of implant development is the attainment of an appropriate drug release profile, which is essential to ensure the desired therapeutic efficacy [19]. However, due to their small dimensions, the intraocular or extraocular implants usually exhibit heightened sensitivity to subtle changes in polymer composition, polymeric matrix ratio, final porosity, and implant geometry. These variations significantly impact surface characteristics, surface‐area‐to‐volume ratio, and drug diffusion pathways, thereby directly influencing release kinetics [20, 21, 22, 23].

Traditionally, the development of the optimal sustained‐release drug delivery systems has relied on empirical design‐of‐experiment methodologies, which require extensive experimental iterations to fine‐tune formulation parameters [24]. However, these trial‐and‐error approaches are time‐consuming and resource‐intensive. Machine learning (ML) models provide a paradigm shift by enabling the predictive modeling of drug release profiles and assisting in the rational design of implant formulations. Unlike conventional methods, ML algorithms can efficiently process complex, nonlinear relationships between material composition, implant structure, and release kinetics, leading to a significant reduction in experimental workload and cost [25, 26, 27]. Moreover, explainable artificial intelligence techniques, such as SHapley Additive exPlanations (SHAP) analysis, can reveal how key formulation parameters influence drug release kinetics, enabling the optimization of sustained‐release implants [28].

**FIGURE 1:**
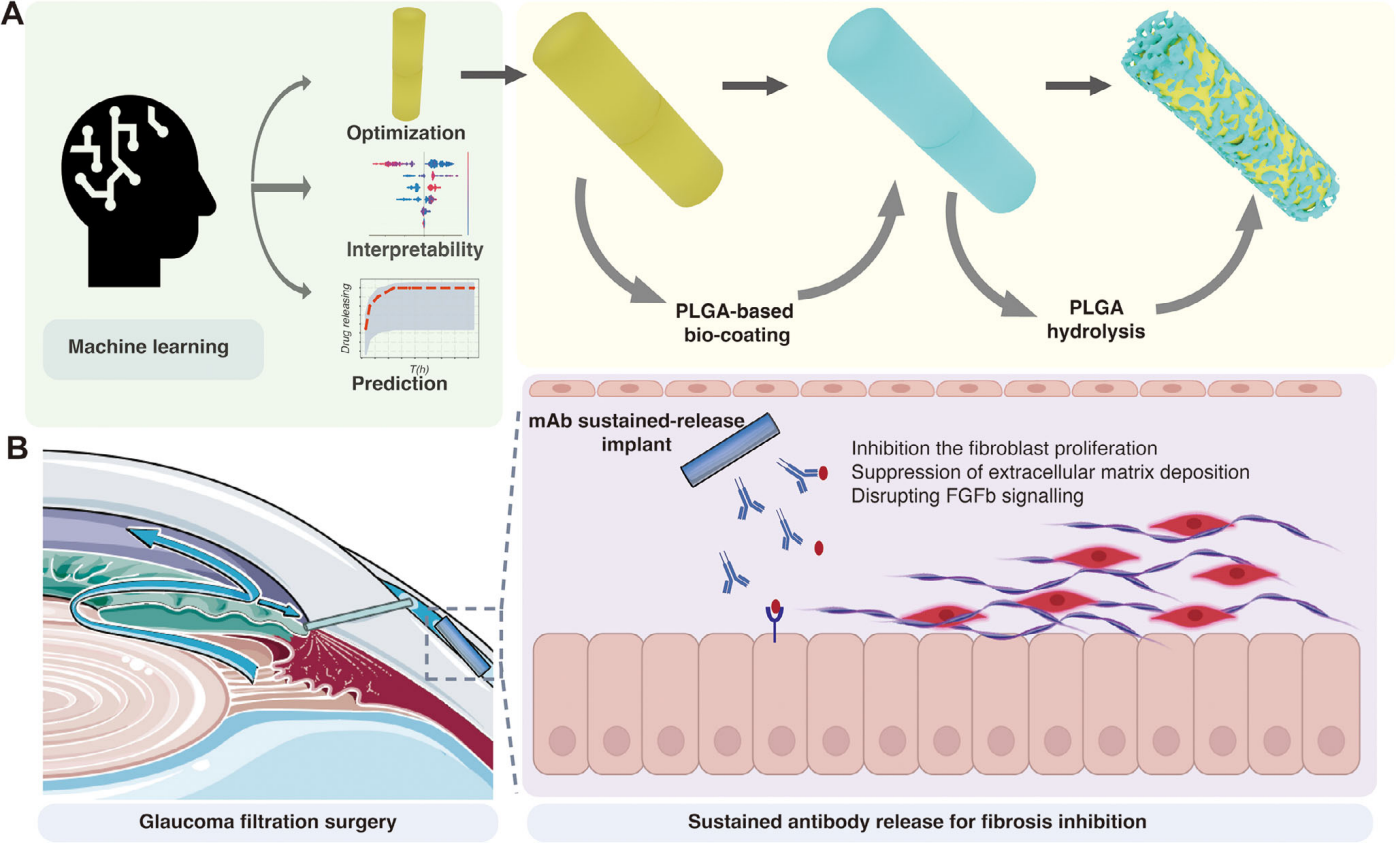
Schematic illustration of machine learning‐assisted optimization of the PCL‐PEG/FGFb mAb@PLGA drug delivery platform for fibrosis inhibition after glaucoma filtration surgery. (A) A machine learning model was used to predict and optimize the drug release profile of PLGA‐based antibody implants, enabling rational design through interpretability and prediction accuracy. The optimized implant was coated with a PLGA shell that undergoes gradual hydrolysis to modulate antibody release kinetics. (B) The implant is placed under the conjunctiva during glaucoma filtration surgery, where it provides sustained monoclonal antibody release to inhibit postoperative fibrosis. Figure 1B was created using BioRender.com.

In this study, we aim to address two critical challenges in the development of ocular drug‐eluting implants. First, the interplay between implant fabrication parameters, such as polymer composition, porosity, and implant dimensions, leads to complex and nonlinear effects on drug release kinetics, making it challenging to rapidly predict release profiles and efficiently screen for optimal formulations [29, 30, 31]. Second, there remains an unmet need for long‐acting anti‐fibrotic implants in glaucoma surgery, for which sustained release technology is essential to modulate conjunctival scarring [32]. To overcome these challenges, we integrated machine learning with polymer science to accelerate the polycaprolactone—polyethylene glycol (PCL‐PEG) implant formulation selection. Our LightGBM model enabled us to (i) decode the multifactorial relationships between implant parameters and drug release profiles, accelerating the prediction of drug release behavior for different implant designs, and (ii) guide formulation selection to maximize mAb delivery efficiency. In addition, we found that the application of a PLGA bio‐coating allowed the PCL‐PEG rod implant to mitigate burst release and to achieve a controlled drug release over an extended period through a dual mechanism of diffusion and polymer degradation. Furthermore, we used FGFb as an anti‐fibrotic target and developed a PCL‐PEG/FGFb mAb@PLGA implant (800 µm in diameter, 3 mm in length) for sustained inhibition of its bioactivity. The PCL‐PEG/FGFb mAb@PLGA implant exhibited efficacy in inhibiting conjunctival fibrosis both in vitro and in vivo (Figure 1). Our findings not only provide strong evidence of the feasibility of using intelligent drug delivery systems in ophthalmology, but also offer a promising therapeutic strategy to improve the surgical outcomes in glaucoma patients.

## 2 Result and Discussion

### 2.1 Implant Fabrication and Drug Release Characterization in PCL‐PEG Systems

**FIGURE 2:**
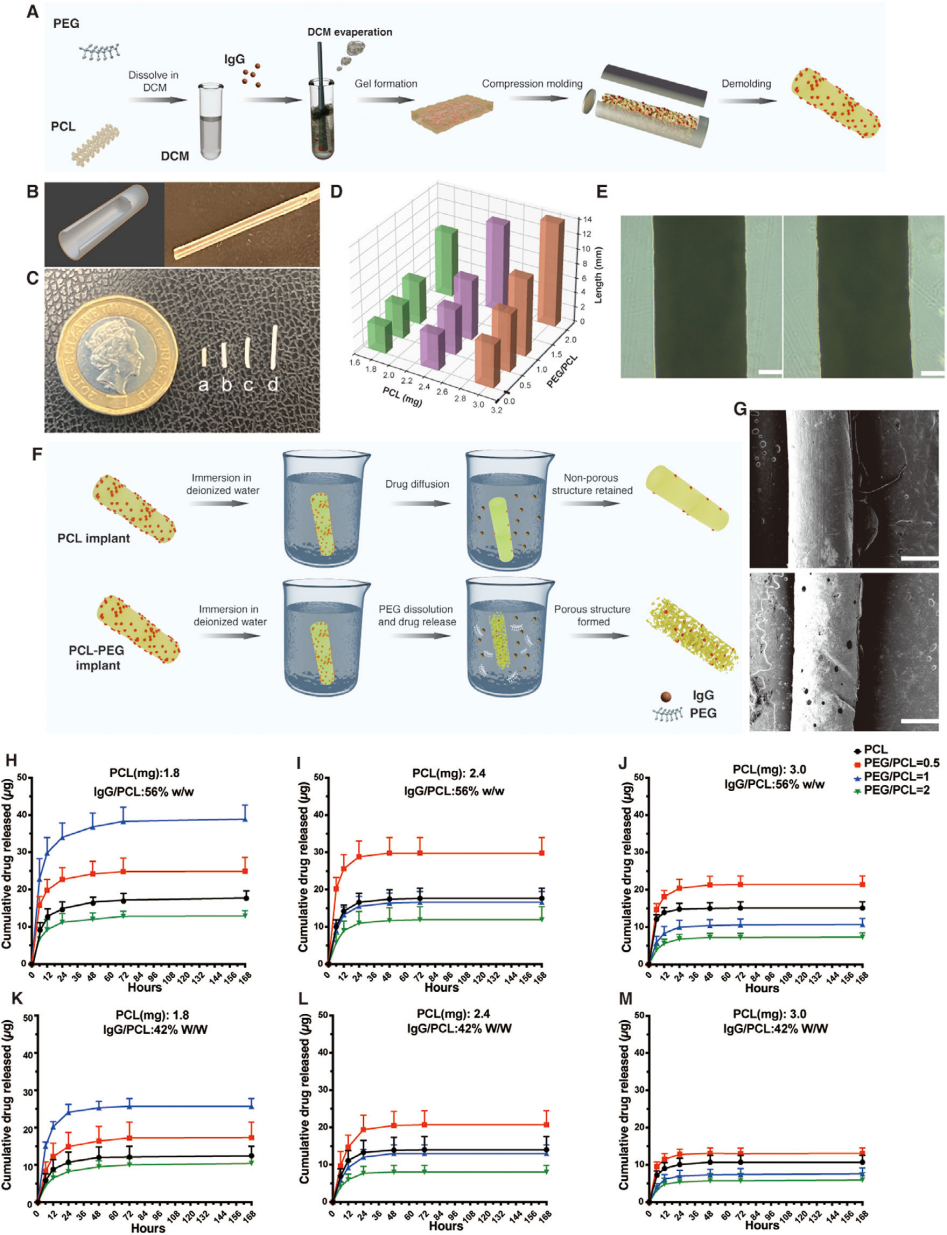
Fabrication and characterization of polymeric implants. (A) Schematic illustration of the implant preparation process. (B) 3D schematic figure and photo of the cylindrical mold used for shaping the implants. (C) Representative images of fabricated implants with varying PCL and PEG/PCL ratios: (a) PCL = 1.8 mg, PEG/PCL = 0; (b) PCL = 1.8 mg, PEG/PCL = 0.5; (c) PCL = 1.8 mg, PEG/PCL = 1; (d) PCL = 1.8 mg, PEG/PCL = 2. (D) Comparison of implant lengths prepared with different PCL and PEG/PCL ratios. (E) Optical microscopy images showing the surface morphology of implants with PCL = 1.8 mg and PEG/PCL = 0 (left) or PEG/PCL = 1 (right). Scale bar = 200 µm. (F) Schematic illustration of the leaching process of drugs and PEG from PCL and PCL–PEG implants upon immersion in deionized water. (G) SEM images revealing the surface morphology of implants with PCL = 1.8 mg and PEG/PCL = 0 (upper) or PEG/PCL = 1 (below) after incubation in deionized water for 7 days. Scale bar = 500 µm. (H) The IgG release profile from the implant (PCL: 1.8 mg, IgG/PCL: 56% w/w). (I) The IgG release profile from the implant (PCL: 2.4 mg, IgG/PCL: 56% w/w). (J) The IgG release profile from the implant (PCL: 3.0 mg, IgG/PCL: 56% w/w). (K) The IgG release profile from the implant (PCL: 1.8 mg, IgG/PCL: 42% w/w). (L) The IgG release profile from the implant (PCL: 2.4 mg, IgG/PCL: 42% w/w). (M) The IgG release profile from the implant (PCL: 3.0 mg, IgG/PCL: 42% w/w). Different lines indicate different PEG/PCL compositions. Black line: the implant composed of PCL only. Red line: PEG/PCL = 0.5. Blue line: PEG/PCL = 1. Green line: PEG/PCL = 2. Results represent mean ± SD, *N* = 10.

The implant was fabricated using a mold‐casting method with polycaprolactone (PCL) and polyethylene glycol (PEG) [33]. During the implant fabrication process, PCL and PEG were first co‐dissolved in dichloromethane (DCM) to form a homogeneous, transparent solution (Figure 2A). The UV–Vis spectral analysis of this liquid phase revealed a characteristic absorbance peak between 220 and 250 nm. Upon DCM evaporation, the system underwent a distinct liquid‐to‐gel transition. The corresponding UV–Vis spectra showed a significant increase in absorbance at 0.1 mm path length, along with pronounced fluctuations and an elevated baseline due to enhanced light scattering, indicating internal structural changes within the polymer matrix (Figure S1).

As depicted in Figure 2B, the high intrinsic viscosity polymer–drug mixture was cast into a cylindrical mold. After peeling off the mold, the fabricated implants exhibited consistent diameters and maintained structural integrity across different formulations (Figure 2C; Figures S2 and S3). However, the addition of varying amounts of PCL and PEG resulted in observable changes in implant length (Figure 2D). Optical microscopy images showed smooth and integrated implant margins without visible cracks or defects, which are essential for consistent drug release and reduced tissue irritation during implantation (Figure 2E).

In this drug delivery system, PEG serves as a porogen due to its water solubility, which allows it to leach out from the PCL matrix upon immersion in an aqueous environment [34]. This leaching process creates a porous structure within the implant, significantly increasing its porosity and facilitating drug diffusion throughout the entire matrix [35, 36], particularly in the deeper layers (Figure 2F). As shown in Figure 2G, after immersion in deionized water for 7 days, a PCL‐only implant retained an intact, non‐porous surface, indicating limited structural change. In contrast, an implant composed of PCL and PEG exhibited clear pore formation, confirming the successful generation of a porous architecture through PEG leaching.

We further investigated the cumulative release profiles of IgG from implants by varying the PCL content, PEG/PCL ratio, and IgG loading (Figure 2H–M). Notably, for implants with the same PCL content and PEG/PCL ratio, those with a higher IgG loading (56% w/w) exhibited a more pronounced initial burst release compared to those with a lower IgG loading (42% w/w), which might be attributed to the increased presence of IgG near the polymer surface, facilitating rapid dissolution upon exposure to the release medium. Regarding the effect of polymer composition on release behavior, the results showed that the drug release profiles did not follow a simple trend with increasing or decreasing amounts of PCL or PEG/PCL ratio. Among the evaluated formulations, the implant containing 1.8 mg of PCL, a PEG/PCL ratio of 1, and an IgG loading of 56% (w/w) exhibited the most sustained release profile. This formulation maintained drug release over 72 h, reaching a cumulative release of 38.3 ± 3.8 µg. These findings highlight the importance of achieving an optimal polymer composition to ensure prolonged drug release.

Besides, the stability of therapeutic antibodies is a critical concern in the manufacturing of drug‐eluting platforms, as antibodies are prone to structural loss, aggregation, and degradation under extreme conditions, such as exposure to organic solvents, mechanical agitation, and fluctuations in temperature or pH [37, 38, 39, 40]. Our study revealed that exposure of IgG to mechanical agitation and DCM during implant fabrication promotes mid‐sized IgG aggregation and light‐chain degradation. The addition of 10% w/v trehalose effectively stabilized the IgG released from the implant by preserving its monomeric form, as evidenced by a particle size distribution similar to that of pure IgG, and maintaining its structural integrity, as demonstrated by the presence of intact heavy and light chains in SDS‐PAGE analysis (Figure S4).

### 2.2 PEG‐Driven Microstructural Transition Enables Water Ingress and Drug Mobility

**FIGURE 3:**
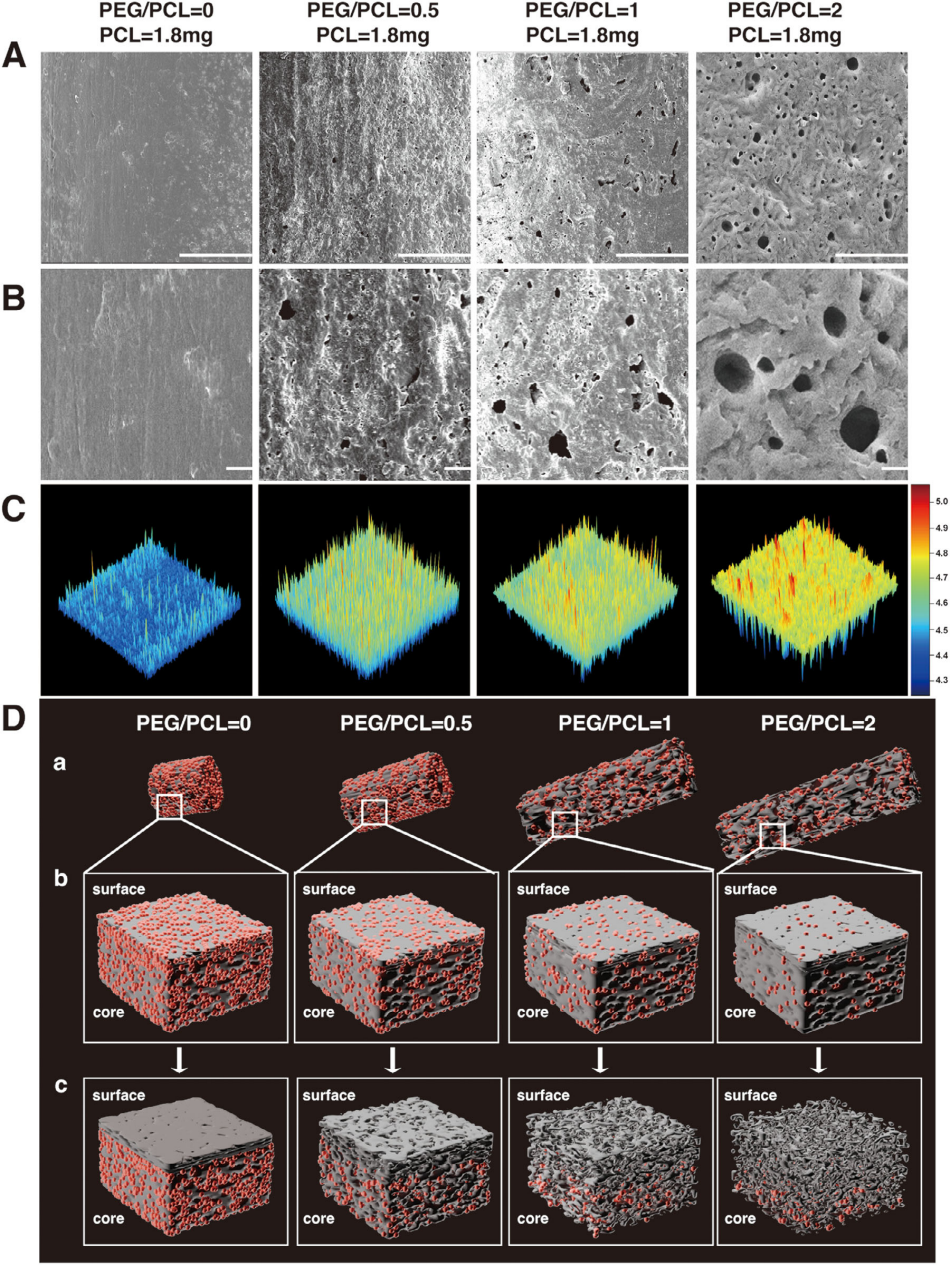
Structural characterization and conceptual modeling of PEG/PCL implants with varying PEG content. (A–B) SEM images showing surface morphologies after 7 days of deionized water immersion. Higher PEG/PCL ratios promote pore formation. Scale bars = 100 µm (A), Scale bars = 10 µm (B). (C) 3D surface roughness plots derived from SEM images, highlighting the increasing surface heterogeneity with higher PEG content. (D) Schematic representation of drug distribution and release behavior. (a) Increasing the PEG/PCL ratio leads to longer implants with lower drug density. (b) Drug packing within a unit volume becomes more diluted as the implant length increases. (c) After water immersion, increasing PEG content leads to more porous structures, which facilitate drug release from the core; however, lower drug density may limit the overall release. Red particles: drug molecules.

In this IgG delivery system, the drug release kinetics exhibited a nonlinear dependence on the contents of PCL and PEG. To explore how polymer composition influences IgG release behavior, we systematically examined the structural and geometric changes in implants with varying PEG/PCL ratios after immersion in deionized water for 7 days. As shown in Figure 3A,B, SEM images revealed that, with PCL fixed at 1.8 mg, increasing PEG content led to progressively more porous morphologies in the implant. While PEG/PCL = 0 implants remained dense and non‐porous, the introduction of PEG (ratios 0.5 to 2) resulted in significant pore formation due to PEG leaching, forming continuous channels that can facilitate drug diffusion. Surface roughness was further visualized using 3D height heat maps reconstructed from SEM images (Figure 3C), confirming the increasing topographical heterogeneity with higher PEG content. These morphological features suggest that PEG functions as a pore‐forming agent, increasing water permeability and allowing drug migration from the implant core to the surface.

However, porosity alone does not fully account for the observed release behavior in our implant system. As shown in Figure S5A, both porosity and implant length increased monotonically with PEG content, with strong correlations for porosity (r = 0.9223) and length (r = 0.9524). This indicates that PEG not only drives pore formation but also contributes substantially to implant elongation. In contrast, varying PCL content (Figure S5B) had minimal influence on porosity (r = 0.0132), yet implant length continued to increase modestly with increasing PCL mass (r = 0.5048), suggesting that PCL primarily affects geometric dimensions rather than pore formation. To further illustrate the interplay between these parameters, Figure S5C represents a 3D distribution of porosity, length, and PEG content, stratified by PCL mass. This visualization highlights that the combined effects of PEG, which increases both porosity and length, and PCL, which mainly increases length, produce distinct structural configurations. These findings demonstrate that polymer composition modulates drug release not only by altering porosity, but also through coordinated changes in geometric parameters, such as implant length.

To interpret the broader implications of these structural changes on drug release kinetics, we developed a schematic model capturing the dual influence of PEG‐induced porosity and length‐dependent geometric dilution (Figure 3D). In Figure 3D‐a, we illustrate that, with a constant PCL mass, an increased PEG/PCL ratio results in a greater polymer mass, which leads to an elongation of the implant when the diameter is constrained. Drug molecules, shown as red particles, become more spatially dispersed as the implant length increases. Figure 3D‐b highlights the drug distribution within a unit volume. Here, implants with lower PEG exhibit compact drug packing, while those with higher PEG demonstrate sparser distribution due to increased internal volume. This dilution reduces the local drug concentration gradient, a critical driver of diffusion‐based release [41]. Figure 3D‐c depicts the residual drug content after water immersion. In low‐porosity implants, most of the drug remains trapped in the core, while higher porosity enables more efficient outward diffusion. However, excessively high porosity often coincides with implant elongation, which dilutes drug distribution and ultimately reduces the effective drug flux due to a lower local concentration gradient.

Together, these findings reveal a mechanistic trade‐off. On one hand, increased porosity enhances release by improving water penetration and drug mobility. On the other hand, increased implant length dilutes drug concentration per unit volume, reducing the driving force for diffusion. Thus, the net release efficiency is governed by a balance between porosity and geometric dilution. These insights underscore the importance of co‐optimizing both composition and implant architecture to achieve the desired release kinetics. Merely increasing PEG to boost porosity may not always enhance drug release if the accompanying geometric expansion excessively dilutes the drug. This design principle is critical in systems with fixed drug doses, where effective implant design can improve the drug utilization efficiency and help maintain the required drug concentration for a sustained therapeutic output.

### 2.3 Machine Learning Assessment to Predict the Drug Release Kinetics

**FIGURE 4:**
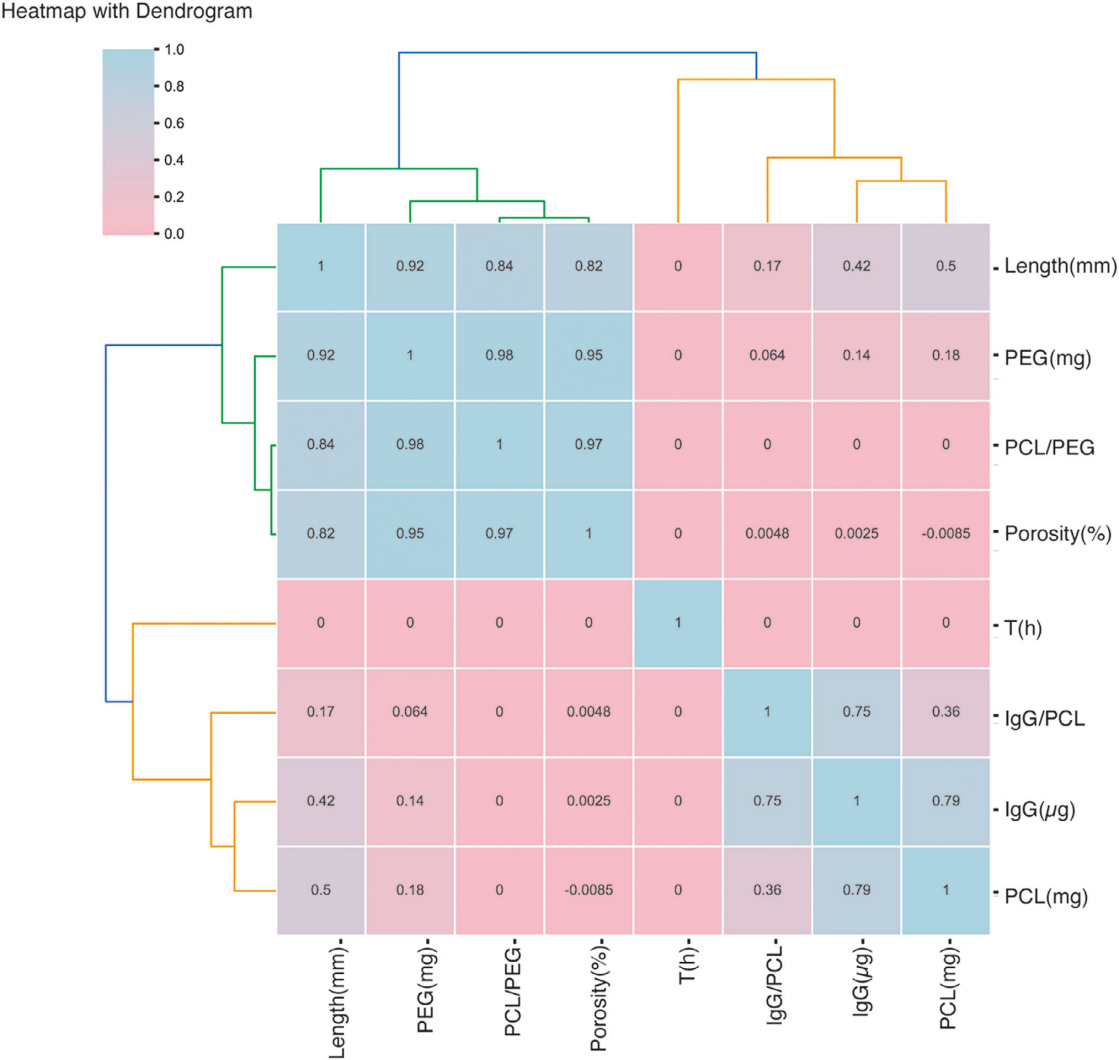
The clustered heatmap with dendrograms of the Spearman correlation between eight selected features: length (mm), PEG (mg), PEG/PCL ratio, porosity (%), IgG (µg), PCL (mg), IgG/PCL, T(h). The red tones represent positive correlations, and the blue tones represent negative correlations. The diagonal exhibits a perfect correlation of 1. Hierarchical clustering has been applied to both rows and columns, resulting in dendrograms that group features with similar correlation patterns.

To facilitate a more efficient understanding of drug release behavior from this polymeric implant system and accelerate the formulation screening process, we employed machine learning (ML) as a powerful modeling tool to predict the drug release profile based on fabrication parameters. ML algorithms are well‐suited for analyzing complex, high‐dimensional interactions, making them particularly effective in evaluating the multifaceted influence of polymer composition on drug release [24]. To train the machine learning model, we initially selected eight input features based on their potential influence on drug release: implant length, porosity, PEG content, PCL content, PEG/PCL ratio, IgG loading, IgG/PCL ratio, and release time. These features were chosen to comprehensively represent the physicochemical properties of the implants, with release time being a critical factor for determining pharmacokinetics. A clustered heatmap with dendrograms was generated to analyze the Spearman correlation between the selected features (Figure 4). Strong correlations were observed between PCL/PEG ratio and PEG (r = 0.98), PCL/PEG ratio and porosity (r = 0.97), as well as PEG and porosity (r = 0.95). To avoid multicollinearity and overfitting, and to ensure that the model variables were primary, controllable, and directly measurable during implant fabrication, the final input set included PCL content, PEG content, IgG loading, implant length, and release time (T). These variables were selected because: (1) PCL, PEG, and IgG are the key formulation components that directly determine drug release; (2) implant length is a controllable and independently measured geometric parameter that influences drug distribution and thereby affects diffusion; and (3) release time is essential for capturing the kinetics of the release process.

**FIGURE 5:**
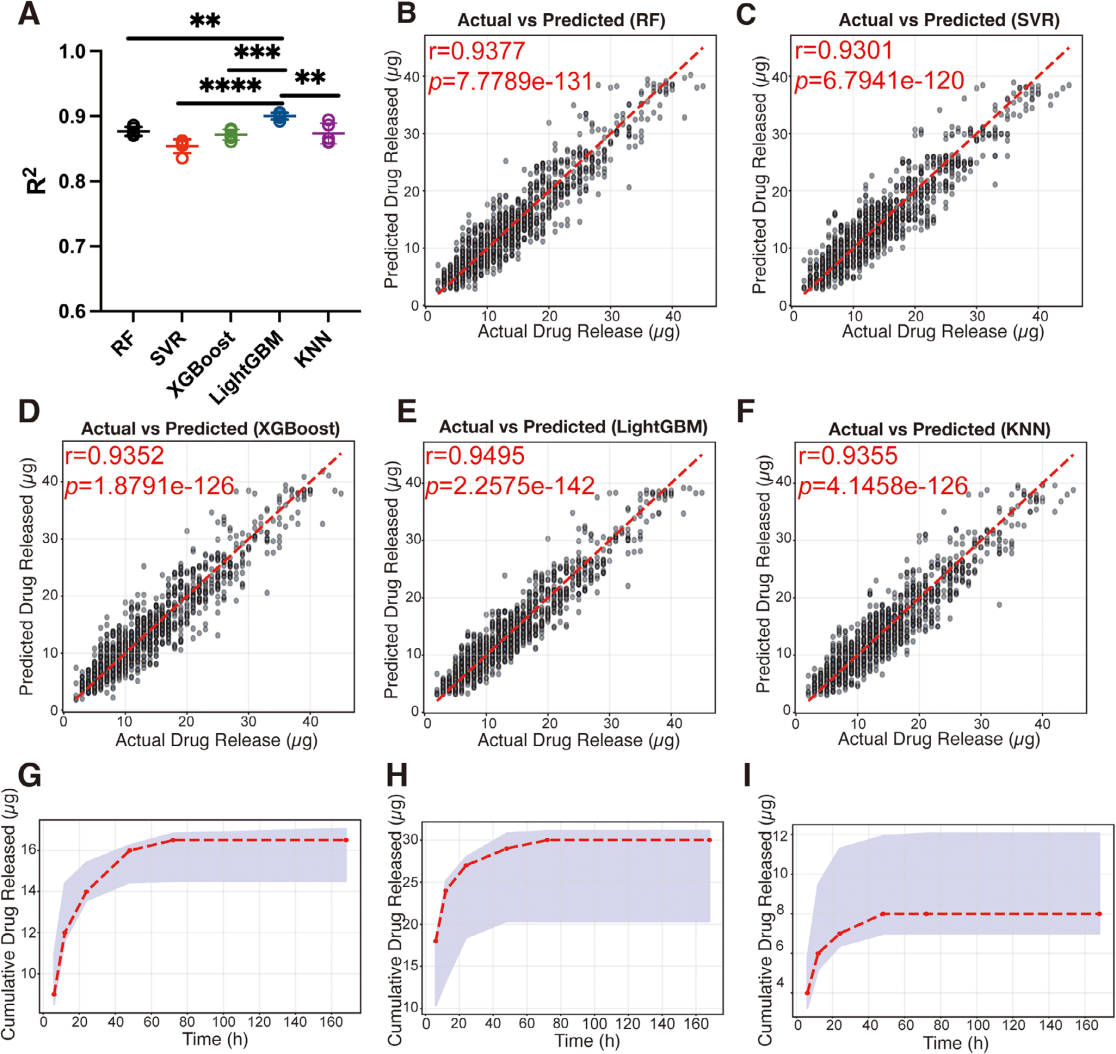
Evaluation of machine learning models for predicting drug release profiles. (A) R^2^ scores for predicted drug release profiles generated by different models (RF, SVR, XGBoost, LightGBM, KNN). (B–F) Comparison of actual and predicted release for RF, SVR, XGBoost, LightGBM, KNN in the outer loop of the nested five‐fold cross‐validation. r: Pearson's r. (G) Comparison of actual data and predicted data for drug release from a new implant by LightGBM. (New implant formulation: PCL = 2.2 mg, PEG = 0 mg, IgG = 123 µg, length = 4.4 mm). (H) Comparison of actual data and predicted data for drug release from a new implant by LightGBM. (New implant formulation: PCL = 2.2 mg, PEG = 1.1 mg, IgG = 123 µg, length = 6.1 mm). (I) Comparison of actual data and predicted data for drug release from a new implant by LightGBM. (New implant formulation: PCL = 2.2 mg, PEG = 4.4 mg, IgG = 123 µg, length = 10.3 mm). **p < 0.01, ***p < 0.001, ****p < 0.0001. One‐way ANOVA with Tukey post hoc test is shown.

We trained five ML models, namely Random Forest (RF), Support Vector Regression (SVR), XGBoost, LightGBM, and k‐Nearest Neighbors (KNN), to predict the drug release behavior. Among the tested machine learning models, LightGBM achieved the highest R^2^ value (0.9000 ± 0.0058) (Figure 5A), along with the lowest mean squared error (MSE, 5.9240 ± 0.2659) and mean absolute error (MAE, 1.8797 ± 0.0430) (Figure S6), thereby demonstrating the best predictive performance for drug diffusion. This can be attributed to LightGBM's leaf‐wise tree growth strategy and efficient handling of small datasets, which enable it to capture intricate patterns and improve model fitting [42]. Scatter plots provide a clear visualization of the alignment between actual and predicted drug release values, further demonstrating the predictive accuracy of each model (Figure 5B–F). The high Pearson's r obtained for all models indicates strong predictive capabilities, with LightGBM achieving the best fit (Pearson's r = 0.9495). This indicates that the LightGBM model successfully captured the underlying relationships between the selected implant parameters and drug release kinetics.

To further evaluate the model's generalizability, we tested the LightGBM model on the implants not included in the training set. The predicted release profile closely aligned with experimental data, including a 90% confidence interval determined by conformal predictions. Overall, the model showed a high reliability in predicting performance with all the actual release data fitting in the prediction interval (Figure 5G–I), thereby demonstrating the model's robustness and potential for real‐world applications.

### 2.4 Machine Learning Interprets the Interaction Between Length, Porosity, and Drug Release

To further quantify the nonlinear relationship between the fabrication parameters of the micro‐cylindrical implant and its drug release profile, we employed SHAP (SHapley Additive exPlanations) analysis, which provides an interpretable framework for complex feature interactions [28]. The SHAP analysis plot ranks the input features by their importance in predicting drug release. Length was identified as the most influential feature, with the highest mean SHAP value (6.2624), followed by PEG (3.0755), time (2.0527), IgG amount (2.0210), and PCL amount (0.6234) (Figure S7).

**FIGURE 6:**
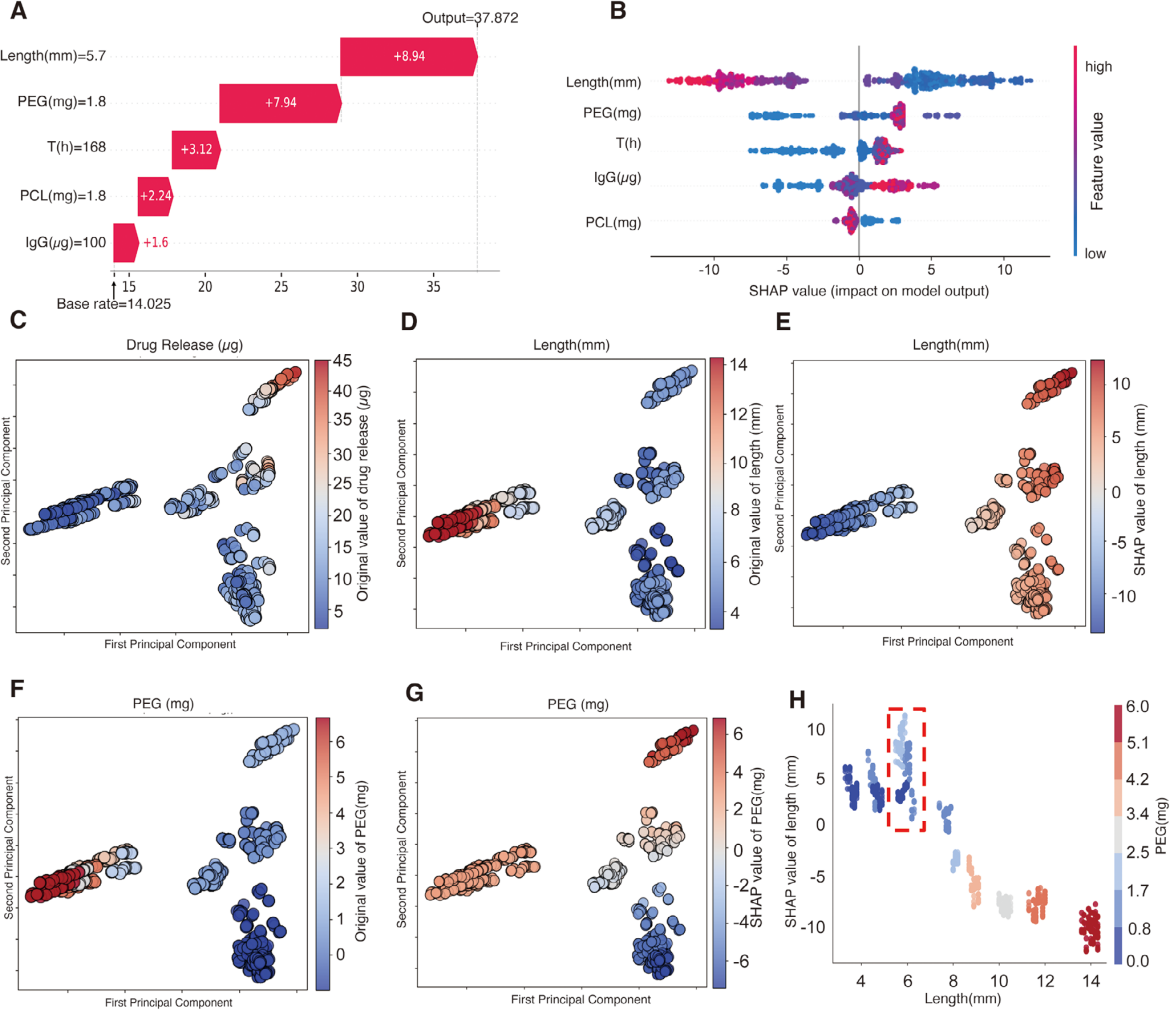
SHAP analysis and principal component visualization of feature contributions to drug release prediction. (A) Feature contribution analysis for LightGBM's prediction, using Implant 3 as an example. (B) SHAP summary plot illustrating the impact of input features on the model output. (C–G) Biplots based on principal component analysis (PCA), highlighting the distribution of length and PEG content and their relationship with drug release. (H) Interplay plot revealing the combined effects of implant length and PEG content on drug release.

To capture the contributions of these key features, an example (Implant 3) of feature contribution to the model's prediction demonstrates how individual input features' content influences the predicted drug release (Figure 6A). For instance, when the length is set to 5.7 mm, the model will increase the predicted drug release by 8.94 µg, reflecting its significant role in modulating the drug release kinetics, followed by PEG content (1.8 mg) at 7.94 µg. The time (168 h) and PCL content (1.8 mg) contribute 3.12 µg and 2.24 µg, respectively, highlighting their role in enhancing drug release. In contrast, the IgG loading (100 µg) only contributes 1.6 µg, indicating its minimal impact.

The SHAP summary plot further illustrates the overall impact and directionality of individual input features on the model's output across the dataset (Figure 6B). It revealed a negative correlation between implant length and SHAP values, indicating that longer implants tend to have a diminished marginal contribution to drug release. In contrast, the relationship between PEG content and SHAP values exhibited a nonlinear pattern. Within a moderate PEG range, a higher PEG content was associated with increased SHAP values, reflecting a greater positive influence on drug release. However, at excessively high PEG levels, SHAP values declined, suggesting that the beneficial effect of PEG was attenuated.

To explore the interaction between structural features and drug release, we performed principal component analysis (PCA). Figure 6C shows that drug release amounts form distinct clusters in the reduced dimensional space, indicating heterogeneous release profiles driven by key variables. Figure 6D reveals a spatial gradient in implant length across clusters, with shorter implants localized in specific PCA regions and longer implants dominating others. This supports the role of implant length as a primary factor influencing the release behavior. SHAP analysis also further corroborates these findings (Figure 6E), showing that shorter implants contribute more positively to drug release predictions, whereas longer implants display progressively lower SHAP values, likely due to diluted drug distribution that limits effective release. The influence of PEG content was also visualized through PCA and SHAP plots. Figure 6F,G reveals a non‐monotonic trend in the relationship between PEG content and SHAP values: PEG content levels within the range of 1–2 mg are associated with the highest SHAP values, indicating optimal conditions for drug release. In contrast, both lower and higher PEG levels correspond to reduced SHAP values, suggesting that either insufficient or excessive PEG content may hinder the drug release efficiency. This pattern can be explained by the feature‐interaction dependency of SHAP values, which quantify the marginal contribution of each feature to the model's prediction, while considering the presence and interaction of all other variables [43]. Although PEG generally enhances drug diffusion by increasing structural permeability, its actual contribution varies depending on other parameters, most notably implant length. In samples where high PEG content coincides with extended implant length, the positive effect of PEG may be attenuated or even reversed due to longer diffusion paths and diminished interfacial flux (Figure 6H). This highlights the importance of accounting for feature interactions when interpreting SHAP‐based insights in drug release modeling.

Temporal dynamics and formulation properties jointly determine the predicted drug release behavior. To disentangle the respective contributions of temporal and formulation features to both predictive performance and interpretability, we performed a feature ablation analysis using five‐fold cross‐validation (Figure S8). The LightGBM models were trained using three input configurations: (i) release time alone, (ii) formulation parameters (length, PEG, PCL, and IgG) alone, and (iii) the combined feature set (length, PEG, PCL, IgG, and release time), which corresponds to the final optimized model used in this study. As shown in Figure S8, the time‐only model exhibited limited predictive capability across all evaluated metrics (R^2^ = 0.0886 ± 0.0236, MSE = 54.1275 ± 3.5793, and MAE = 5.6675 ± 0.3121), indicating that release time alone captures a weak global temporal trend and fails to account for the substantial inter‐formulation variability in release behavior. In contrast, the formulation‐only model achieved markedly higher predictive performance (R^2^ = 0.7796 ± 0.0161, MSE = 13.0670 ± 0.8386, and MAE = 2.7666 ± 0.0992), demonstrating that formulation parameters provide the primary predictive information used by the model. The highest predictive accuracy was obtained using the final model that incorporated both release time and formulation parameters (R^2^ = 0.9000 ± 0.0058, MSE = 5.9240 ± 0.2659, and MAE = 1.8797 ± 0.0430). These results indicate that release time is required to describe the temporal progression of drug release, whereas formulation parameters primarily determine the magnitude and variability of the model‐predicted release across the release period. In addition, SHAP analysis consistently shows that formulation parameters collectively dominate feature importance (mean SHAP values: Length = 6.2624, PEG = 3.0755) compared to the release time parameter (2.0527) (Figure S7), further reinforcing that formulation‐level features primarily drive both predictive performance and model interpretability.

Together, these results underscore the importance of considering the combined effects of implant length and PEG in the design and optimization of drug delivery systems. By accounting for the interplay between these parameters, it is possible to mitigate the over‐reliance on a single parameter and to ensure the stability and controllability of drug release performance.

### 2.5 Machine Learning Optimizes Implant Formulation

**FIGURE 7:**
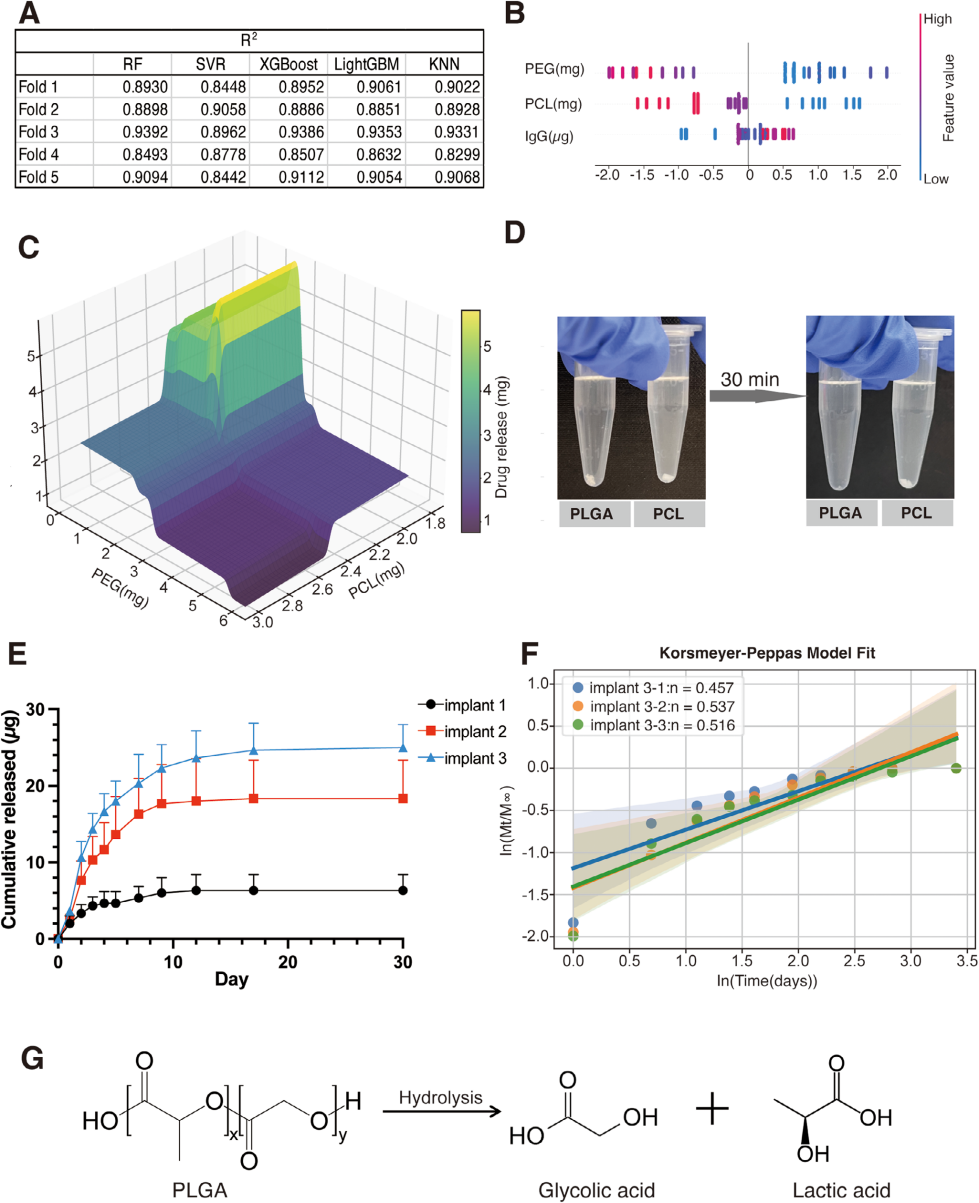
Preparation of a sustained IgG‐releasing PCL‐PEG@PLGA implant with decreased burst release. (A) Performance comparison of five machine learning models (RF, SVR, XGBoost, LightGBM, KNN) in predicting drug release amounts per millimeter of implant length over 7 days. (B) SHAP summary plot illustrating the contribution of individual features to the LightGBM model's predictions. (C) A three‐dimensional surface plot illustrating the combined effects of PEG and PCL concentrations on drug release per millimeter of implant length, as predicted by the LightGBM model. (D) Comparative dissolution of PLGA and PCL in DMSO (0.1% w/v) after 30 min. (E) Cumulative drug release profiles for PCL‐PEG implants (3 mm) with and without PLGA coatings over a 30‐day period. Results represent mean ± SD, *N* = 3. (F) The logarithmic relationship between time (days) and cumulative drug release ratio (𝑀𝑡/𝑀∞) fitted using the Korsmeyer–Peppas model. (G) Chemical equation for the hydrolysis of PLGA.

As previously mentioned, optimal release profiles emerged when the incorporation of moderate polymers balanced pore connectivity along with restrained implant elongation, allowing porous interface expansion to offset distribution dilution. The PCL and PEG interplay establishes a critical formulation threshold where structural accessibility and drug density achieve kinetic equilibrium, requiring precise stoichiometric control of polymer components. Then, to develop a machine learning framework for optimizing implant preparation parameters, we trained five distinct ML models using the following input features: (1) PCL concentration, (2) PEG concentration, and (3) IgG loading amount. We chose PCL and PEG because their amounts are easily modified during pharmaceutical fabrication. The target output was the cumulative drug release amount per millimeter of implant length over 7 days. Among these models, LightGBM demonstrated superior predictive performance, achieving the highest R^2^ (0.8996 ± 0.0258) (Figure 7A), the lowest MSE (0.3142 ± 0.0797) (Table S1), and the lowest MAE (0.3840 ± 0.0286) (Table S2). We performed SHAP analysis to elucidate the factors driving the model's predictions. The results revealed that PEG and PCL were the most significant contributors to drug release predictions, with mean SHAP values of 1.1929 and 0.7735, respectively. The IgG ratio also played a notable role (0.3355) (Figure 7B and Figure S9).

Using the trained LightGBM model, we generated a 3D prediction surface to simulate drug release per millimeter of implant length over 7 days. The simulation was performed with a fixed IgG input of 100 µg to ensure consistency across varying PEG and PCL compositions. Under this fixed IgG input, the model predicted drug release performance across a range of PCL amounts and PEG/PCL ratios. The optimal formulation range was identified as 1.800–2.373 mg PCL and 1.688–1.960 mg PEG. Within this window, the predicted release reached 95% of the maximal response, corresponding to 5.119–5.604 µg/mm (Figure 7C).

Guided by these predictions, we fabricated cylindrical implants containing 1.8 mg of PCL and 1.7 mg of PEG, loaded with 100 µg of IgG. Due to the unique microstructures of the eye, the size of an ocular implant must be carefully constrained. The implants were then segmented into 3 mm‐long implants. To mitigate burst release, the implants were coated with 0.1% w/v PLGA solution (dissolved in DMSO). Importantly, PLGA is soluble in DMSO [44], whereas PCL remains insoluble, ensuring that the bio‐coating provides sustained release without compromising the structural integrity of the PCL‐based implants (Figure 7D). The cumulative drug release profiles of 3 mm‐long PCL‐PEG implants, both with and without PLGA coating, were evaluated over 30 days to compare their sustained‐release performance. The in vitro drug release study revealed significant differences between uncoated and PLGA‐coated PCL‐PEG implants over 30 days. Uncoated implants exhibited rapid burst release, achieving 73.58 ± 9.38% release within the first day and reaching complete drug elution by days 4–5. In contrast, PLGA‐coated implants demonstrated sustained‐release kinetics, with only 35.04 ± 3.91% released by day 3, 57.26 ± 7.70% by day 9, and gradually reaching 64.10 ± 7.69% cumulative release by day 30. The coating significantly attenuated the burst effect while extending the release duration, and the results clearly demonstrated the ability of the PLGA coating to modulate the drug release profiles by retarding the initial release and by prolonging the sustained delivery (Figure 7E).

To investigate the sustained‐release mechanism of the PCL‐PEG/PLGA implant, we applied the Korsmeyer–Peppas model to the release data. The results indicated a non‐Fickian release mechanism (n = 0.50 ± 0.04), suggesting that the drug release was driven by a combination of diffusion and polymer degradation (Figure 7F) [25, 45]. The polymer degradation is likely due to the PLGA bio‐coating, which can hydrolyze rapidly into nontoxic components (glycolic acid and lactic acid) (Figure 7G) [46]. This degradation provides a controlled and biocompatible drug release mechanism, making PLGA an ideal coating material for ocular implants.

### 2.6 Efficacy of PCL‐PEG/FGFb mAb@PLGA Implants In Vitro and In Vivo

Basic fibroblast growth factor (FGFb) has been identified as a potential therapeutic target to inhibit fibrosis in glaucoma filtration surgery [32, 47]. Increasing evidence highlights its critical role in fibrosis pathogenesis, as FGFb is markedly upregulated in fibrotic tissues [48] and has been implicated in driving conjunctival fibrosis in both human and rabbit models [49, 50]. Mechanistically, it also synergizes with a network of profibrotic mediators, including TNF‐α, TGF‐β, EGF, and PDGF, to amplify pathological processes, such as fibroblast transdifferentiation, migration, and extracellular matrix (ECM) remodeling [51, 52]. Supporting this, our transcriptomic profiling demonstrated that *FGFb* consistently exhibited higher expression levels than *TGF‐β2* across aqueous shunt implantation sites [53] and conjunctival subtypes [54] (Figure S10A,B). Additionally, the protein–protein interaction network revealed strong associations between FGFb and key fibrosis‐related proteins, including TGF‐β1, FN1, and VEGFa (Figure S10 C) [55, 56, 57]. Collectively, these findings highlight FGFb's potential as a key target for conjunctival anti‐scarring therapy. In this research, we thus used FGFb mAb as a therapeutic agent for modulating post‐surgical fibrosis.

**FIGURE 8:**
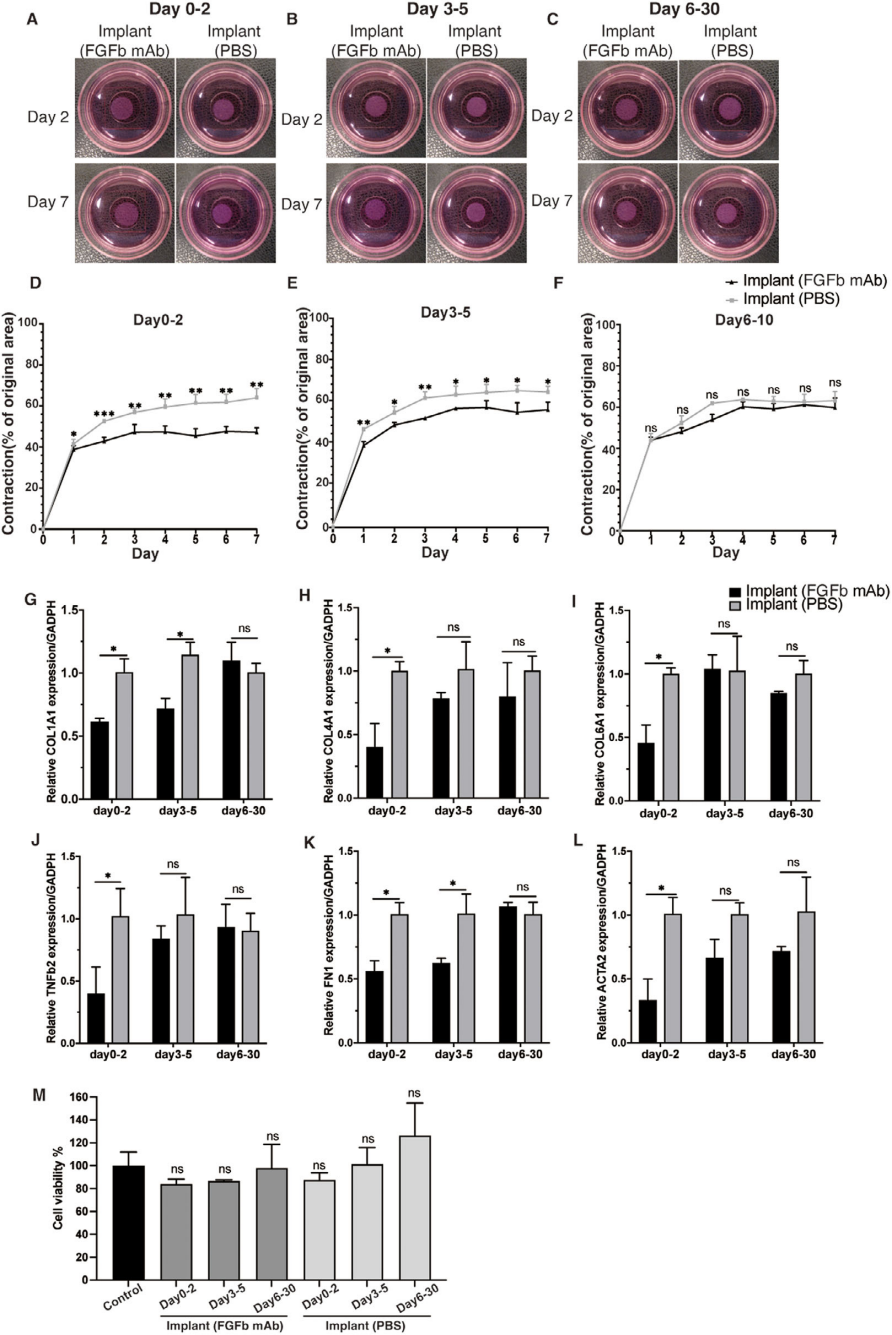
Anti‐fibrotic effect of the PCL‐PEG/FGFb mAb@PLGA implant on rat conjunctival fibroblasts. (A–C) Seven‐day collagen contraction assay of rat conjunctival fibroblasts after treatment with the drug solutions of the PCL‐PEG@PLGA implant with and without FGFb antibody collected from (A) days 0–2, (B) days 3–5, (C) days 6–30. Representative gel images on day 2 and day 7 are shown. (D–F) The percentage of collagen contraction from day 1 to day 7 was calculated from the original area. (G–L) Gene expression of (G) *COL1A1*, (H) *COL4A1*, (I) *COL6A1*, (J) *TGF‐β2* (K) *FN1* and (L) *ACTA2* in rat conjunctival fibroblasts after 2‐day treatment with drug solutions of the PCL‐PEG@PLGA implant with and without FGFb antibody collected from days 0–2, days 3–5, and days 6–30, respectively. The mRNA expression was normalized against *GAPDH*. (M) Cell viability of rat conjunctival fibroblasts after treatment with the drug solutions of the PCL‐PEG@PLGA implant with and without FGFb antibody. Results represent mean ± SD, *N* = 3. ns, not significant; *p < 0.05; **p < 0.01; ***p < 0.001. Student's unpaired *t*‐test was used for gel contraction analysis and qPCR analysis. One‐way ANOVA with Tukey post hoc test was used for cell viability analysis.

To evaluate the temporal efficacy of PCL‐PEG/FGFb mAb@PLGA implant over time, we performed a collagen gel contraction assay (days 0–2, days 3–5, days 6–30 eluates) (Figure 8A–C). Quantitative analysis demonstrated phase‐dependent, anti‐contractile effects (Figure 8D–F). Days 0–2 eluates achieved the maximal inhibition, reducing the contraction area by 15.5% versus PBS controls at Day 7 (*p* = 0.002). This potency declined gradually, with days 3–5 eluates showing 8.6% inhibition (*p* = 0.031) and days 6–30 eluates exhibiting no significant differences compared to controls.

Then, gene expression profiling provided mechanistic insights into the anti‐fibrotic effects of the PCL‐PEG/FGFb mAb@PLGA implant in different phases (Figure 8G–L). Compared to the implant loaded with PBS, early‐phase eluates (days 0–2) of the implant loaded with FGFb antibody elicited a robust transcriptional response, characterized by a decreased gene expression of *COL1A1* (*p* = 0.011), *COL4A1* (*p* = 0.013), *COL6A1* (*p* = 0.015), *TGF‐β2* (*p* = 0.013), *FN1* (*p* = 0.018), and *ACTA2* (*p* = 0.017). Intermediate‐phase eluates (days 3–5) of antibody‐loaded implant exhibited a sustained downregulation of *COL1A1* (*p* = 0.015) and *FN1* (*p* = 0.015), while other targets trended toward baseline expression. By days 6–30, no significant differences in gene expression were observed compared to PBS controls. In addition, cell viability assays confirmed preserved cell viability across all treatment conditions (Figure 8M), demonstrating the elution process maintained antibody biocompatibility without cytotoxic by‐product generation.

**FIGURE 9:**
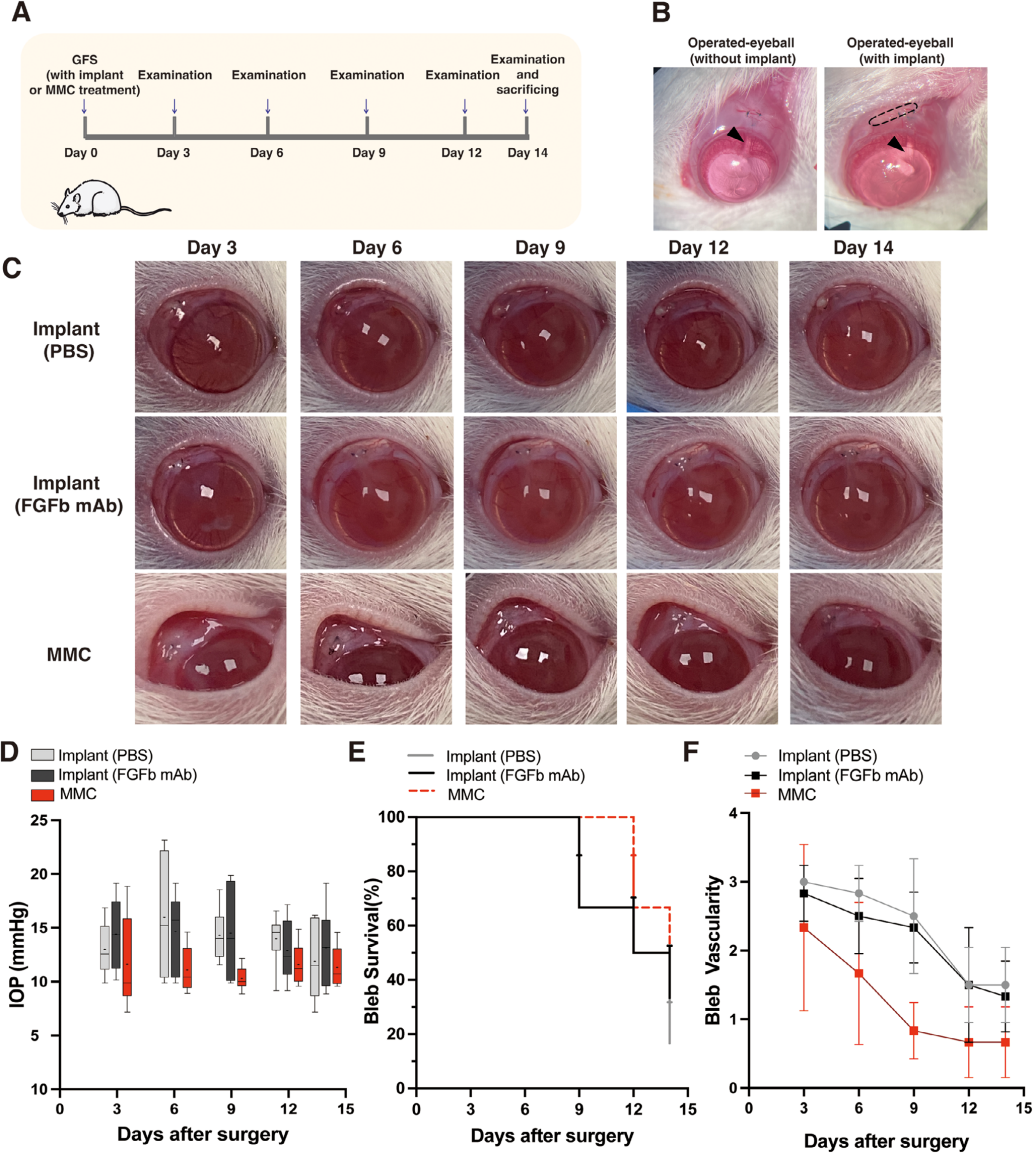
Anti‐fibrotic effect of the PCL‐PEG/FGFb mAb@PLGA implant in a rat model of glaucoma filtration surgery (GFS). (A) Schematic diagram to illustrate the experimental protocol for the rats after GFS. (B) Arrowheads indicate the glaucoma filtration tube; the black line indicates the outline of the implant. (C) Postoperative bleb morphology was evaluated in the eyes treated with the PCL‐PEG@PLGA implant (with and without FGFb antibody) and with MMC treatment (D) IOP level, (E) bleb survival, (F) bleb vascularity (0: avascular; 1: normal; 2: hyperaemic; 3: very hyperaemic) between the PCL‐PEG@PLGA implant (with FGFb antibody and without FGFb antibody) and the MMC‐treated group. Results represent mean ± SD, *N* = 6.

We next evaluated the biocompatibility and potential toxicity of the PCL‐PEG/FGFb mAb@PLGA implant in a rat model of glaucoma filtration surgery (GFS), with PCL‐PEG/PBS @PLGA implant and mitomycin C (MMC) treatment serving as controls. We monitored conjunctival and corneal toxicities, anterior chamber inflammation, bleb morphology, and intraocular pressure (IOP) over a 14‐day period. The experimental timeline involved examinations at days 3, 6, 9, 12, and 14 post‐surgery (Figure 9A). Representative images showed the morphology of the rat eye with and without the implant right after the completion of glaucoma surgery, and highlighted the position of the filtration tube and the placement of the implant (Figure 9B). Morphological assessment of the filtering bleb demonstrated a progressive reduction in bleb size over time in both the implant‐treated groups as well as the MMC‐treated group (Figure 9C).

Throughout the experimental period, all three groups (PCL‐PEG/FGFb mAb@PLGA implant, PCL‐PEG/PBS@PLGA implant, and MMC) exhibited no signs of bleb leakage, anterior chamber (AC) inflammation, conjunctival or corneal toxicity, or systemic adverse effects. These safety profiles suggest that the PCL‐PEG/FGFb mAb@PLGA implant group, which involved sustained antibody release, did not display high‐dose off‐target effects. All rats survived to the endpoint (day 14), underscoring the implant's safety and potential for clinical translation. Functional assessments of IOP revealed that it remained within normal ranges in all three groups throughout the study, indicating unimpaired drainage and no significant tube blockage (Figure 9D). No significant differences in bleb survival were observed among the implant (FGFb mAb), implant (PBS), and MMC‐treated groups. By the study endpoint (day 14), the bleb survival was maintained in 2 rats in both the FGFb mAb group and the MMC group, and in 1 rat in the PBS group (Figure 9E). Vascularity scores declined progressively in the PBS and FGFb mAb implant groups, with no significant differences between them. By day 14, vascularity scores in both groups were close to the normal baseline value of 1 (PBS implant: 1.5 ± 0.55; mAb implant: 1.33 ± 0.52), indicating that early vascular proliferation was a transient postoperative response that resolved naturally, returning to a near‐normal state, and that neither implant induced long‐term inflammation. In contrast, MMC treatment exerted a marked inhibitory effect on conjunctival vascularity, resulting in an avascular state in 2 out of 6 eyes by day 14 (vascularity score: 0.67 ± 0.52) (Figure 9F). This observation aligns with its well‐characterized cytotoxic and anti‐angiogenic mechanisms, and can lead to sight‐threatening complications, such as bleb leakage and endophthalmitis [58]^.^ These results indicate that although the antifibrotic efficacy of the FGFb mAb implant, as reflected by bleb survival, is comparable to that of MMC, it does not induce the pronounced vascular toxicity observed with MMC. The preservation of a more physiological conjunctival vascularity in the FGFb mAb group may therefore represent an early safety advantage over the more aggressive vascular suppression associated with MMC. Furthermore, the ability of the sustained‐release FGFb mAb implant to achieve significant early fibrosis control while maintaining healthier vascular architecture highlights its potential as a safer and more clinically sustainable alternative to MMC.

**FIGURE 10:**
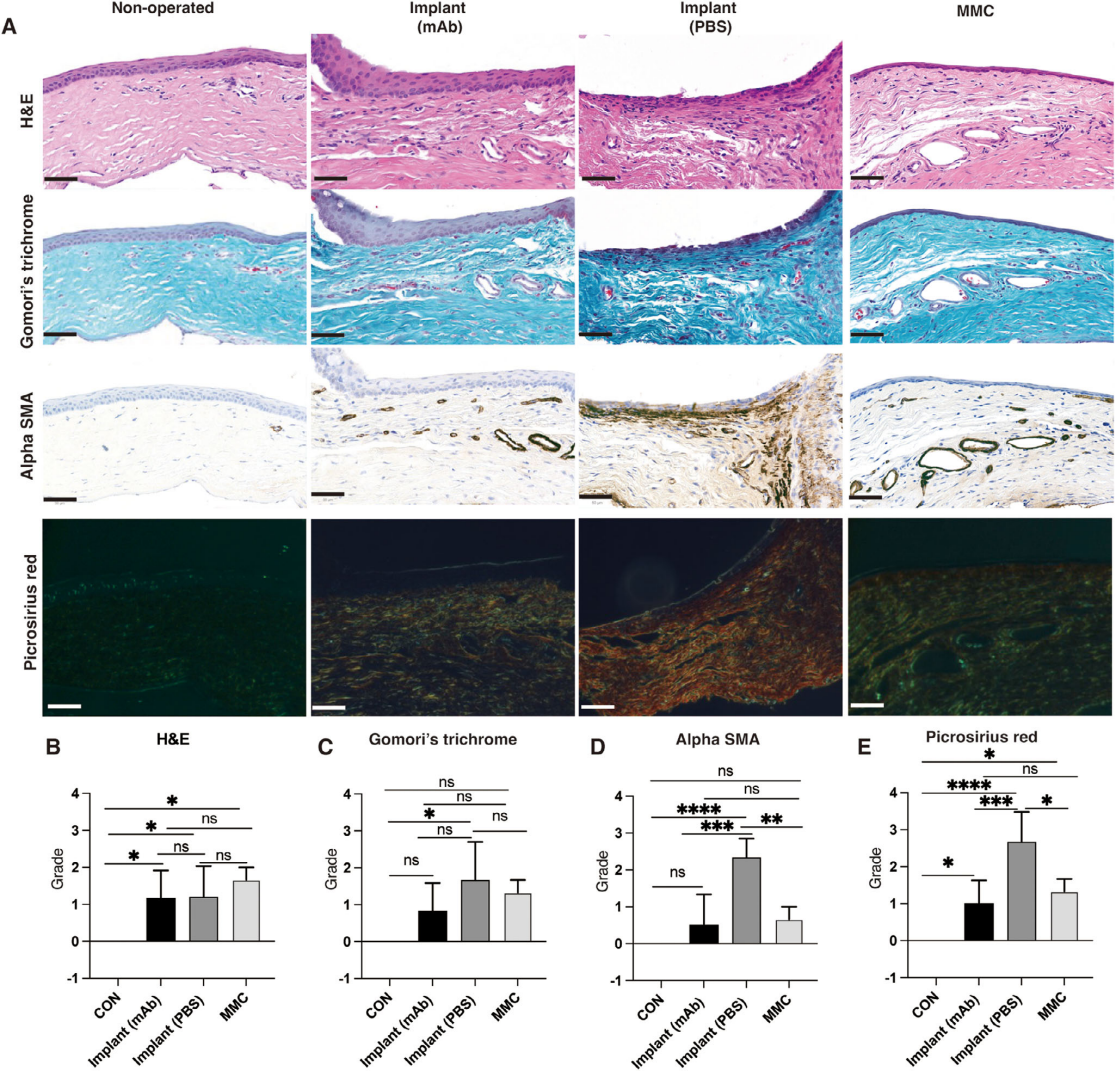
Histology of rat conjunctival tissues. The right operated eyes were compared to the left non‐operated eyes that were used as controls for normal conjunctival tissue. (A) H&E, Gomori's trichrome, αSMA, and Picrosirius red staining. Scale bars = 50 µm. (B–E) Statistical analyses of the grades of H&E, Gomori's trichrome, αSMA, and Picrosirius red staining in non‐operated eyes, the operated eyes with FGFb mAb implant, the operated eyes with PBS implant, and the operated eyes with MMC treatment. Results represent mean ± SD, *N* = 6. ns, not significant; *p < 0.05; **p < 0.01; ***p < 0.001; ****p < 0.0001. One‐way ANOVA with Tukey post hoc test is shown.

We then investigated the antifibrotic effects of the PCL‐PEG@PLGA implant, either loaded with FGFb antibody or with PBS, as well as the MMC‐treated group, on conjunctival wound healing after the 14‐day postoperative period. The implant (PBS) group exhibited significant inflammation and fibrosis, with H&E staining, revealing irregular tissue architecture and increased cellularity. Gomori's trichrome and Picrosirius red staining demonstrated elevated collagen deposition, and α‐SMA immunohistochemistry showed increased myofibroblast activation (Figure 10A). In contrast, both the implant (FGFb mAb) and MMC groups exhibited comparable anti‐fibrotic efficacy, as evidenced by markedly reduced collagen deposition and myofibroblast activation compared to the implant (PBS) group (Figure 10B–E). Collectively, these findings suggest that the PCL‐PEG/FGFb mAb@PLGA implant effectively decreases postoperative scarring by suppressing fibroblast activation and collagen deposition, highlighting its potential to improve outcomes in glaucoma filtration surgery.

Our histological findings demonstrate that the PCL‐PEG/FGFb mAb@PLGA implant suppresses fibroblast activation and collagen deposition to a degree that matches the efficacy of mitomycin C, the current clinical gold standard regimen [59]. This antifibrotic effect can be attributed to a release strategy that is aligned with the pathological progression of conjunctival scarring. In the rat model of GFS, conjunctival scarring formation is characterized by an initial rapid acute inflammatory phase (days 0–2), followed by an active proliferative phase (days 3–10), and a prolonged remodeling phase (beyond day 10) [60]. The initial inflammatory and proliferative phases represent a period of intense fibrotic activity. During this time, peak inflammatory cell infiltration, profibrotic mediator expression, and extracellular matrix accumulation occur, necessitating high‐dose drug release to suppress rapid matrix deposition and to prevent granulation tissue formation [60, 61]. The remodeling phase is marked by a residual, low‐level fibrogenic signal from macrophages [60, 62]; accordingly, a sustained low‐dose release of FGFb mAb is sufficient to prevent excessive scar maturation without disturbing the immune homeostasis or causing tissue toxicity. Our implant facilitated the release of the FGFb mAb through a non‐Fickian diffusion mechanism (Korsmeyer–Peppas, n = 0.50 ± 0.04), which was characterized by an initially high flux that subsequently decelerated in accordance with a power‐law function [63]. This profile delivered an early high drug flux during the first 10 days, aligning with the period of peak fibrotic activity, and transitioned to a sustained low‐level release throughout the remodeling phase. This temporal alignment attenuates fibrosis and avoids potential tissue toxicity, representing a promising antifibrotic strategy following GFS. It should be noted that fibrosis develops rapidly in the rat GFS model with bleb failure typically occurring within 8–13 days due to aggressive fibroproliferation [64, 65], The anti‐fibrotic effect of our FGFb mAb implant was thus evaluated during the early postoperative period of 14 days in the rat GFS model, which encompasses the acute inflammatory phase (days 0–2), the proliferative phase (days 3–10), and the early remodeling phase (beyond day 10) [60, 61], and therefore the period during which the released antibody is expected to exert its maximal antifibrotic efficacy. The 14‐day follow‐up in the rat GFS model captures the peak phase of fibroblast activation and ECM deposition, which are the mechanistic targets of FGFb inhibition, but cannot reflect the long‐term bleb survival, fibrosis progression, or late safety events observed clinically. To address this limitation, future studies using large‐animal trabeculectomy models with slower wound‐healing kinetics are planned to evaluate the sustained and long‐term antifibrotic efficacy of the implant over several weeks to months.

To advance this platform toward clinical translation, future work will focus on two complementary directions. First, in this study, we used a rat model of GFS to provide proof‐of‐concept evidence for the pharmacokinetic profile and antifibrotic performance of the sustained‐release FGFb mAb implant. Future work will involve validating the implant in a classic trabeculectomy model in larger animals, such as rabbits or non‐human primates, which better replicate the human surgical anatomy, postoperative wound‐healing responses, and clinical challenges (e.g., episcleral fibrosis, bleb morphology, and aqueous outflow dynamics). Besides, non‐human primates, such as rhesus monkeys, offer additional advantages, including the ability to induce glaucoma and to perform surgery on eyes with elevated IOP, providing pathophysiological conditions that more closely resemble those of the human glaucomatous eye and enabling a more clinically and translationally relevant evaluation of the implant‘s efficacy and postoperative outcomes [66, 67, 68]. Second, the machine learning framework could be extended from a forward predictor to a rigorous inverse‐design tool. This would involve a two‐step process: first, using ML to predict key parameters (e.g., k and n) of a mechanistic release model from formulation inputs, and second, employing Bayesian optimization to identify optimal formulations for predefined target release profiles. These computational strategies could facilitate the rational design of next‐generation ocular implants.

## 3 Conclusion

In this study, we developed a novel drug‐eluting implant platform (PCL‐PEG/FGFb mAb@PLGA) by integrating polymer engineering, machine learning, and targeted anti‐fibrotic therapy to address the critical challenges of postoperative fibrosis and controlled drug release. Using machine learning models, particularly LightGBM, we successfully predicted the drug release profiles, identified the implant formulation achieving the maximal release, and elucidated the combined roles of implant length, porosity, and polymer composition in governing the release behavior. This ML‐driven strategy not only enhanced our understanding of the release mechanisms, but also enabled the design of implants with tailored drug delivery profiles. Besides, the implant platform effectively protects monoclonal antibodies from denaturation during manufacturing and enables sustained antibody release through a synergistic combination of diffusion and polymer degradation mechanisms. Furthermore, the PCL‐PEG/FGFb mAb@PLGA implant demonstrated significant anti‐fibrotic efficacy in both in vitro and in vivo models. In a rat model of GFS, it demonstrated good tissue biocompatibility and no toxicity, highlighting its potential to transform postoperative care through sustained, localized drug delivery with minimal adverse effects.

Our work represents a significant advancement in the field of ocular drug delivery, addressing the limitations of conventional implant design strategies. By integrating material science and computational modeling, this study paves the way for the development of next‐generation implants with enhanced therapeutic outcomes and clinical applicability.

## 4 Experimental Section

### 4.1 Materials

Polycaprolactone (PCL) (Mw = 50,000 Da) (25090‐100) was purchased from Generon (Slough, UK), polyethylene glycol (PEG) 3350 (25322‐68‐3) was purchased from VWR international (Leicestershire, UK), and poly (lactic‐co‐glycolic acid) (PLGA) (50:50) (Mw = 10 000–20 000 Da) (34346‐01‐5) was purchased from Cambridge Bioscience (Cambridge, UK). IgG (I4506), dichloromethane (DCM) (75‐09‐2), dimethyl sulfoxide (DMSO) (D4540), D‐(+)‐trehalose dihydrate (T9531), and penicillin–streptomycin (P4333) were purchased from Merck Sigma (Dorset, UK). NeutraKineFGF basic Monoclonal antibody (69024) was purchased from Proteintech (Manchester, UK). Phosphate‐buffered saline (11593377) was bought from Fisher Scientific (Leicestershire, UK). Dulbecco's modified Eagle's medium (DMEM) (41966‐029), L‐glutamine (25030‐024), Trypsin (25300‐054), and fetal calf serum (FCS) (A5256701) were purchased from Thermo Fisher Scientific (Cheshire, UK).

### 4.2 Preparation of the Micro‐Cylinder Implant

The PCL and PEG were dissolved in 70 µL DCM. Subsequently, 5 µL of a drug solution (IgG or FGFb antibody dissolved in PBS and supplemented with 10% w/v trehalose) was added to the polymer mixture. The drug‐loaded mixture was aspirated at a flow rate of 210 µL/sec for 6 s to ensure homogeneity. It was then stirred for 15 s to facilitate the evaporation of DCM at room temperature. As the DCM evaporated, the mixture gradually transitioned into a drug‐incorporated gel. The drug‐incorporated gel was pressed into an 800 µm diameter cylindrical mold under an applied pressure of 1.17 MPa and allowed to solidify at room temperature for 2 h. Once solidification was complete, the mold was carefully peeled away to obtain the final implant with a diameter of 800 µm. All procedures were performed in a sterile fume hood. The masses of PCL, PEG, and drug concentration used for each implant, along with their mass ratios, are provided in Table 1.

**TABLE 1:** Compositions and parameters of the different implants.

Implant ID	IgG (µg)	IgG/PCL w/w%	PCL (mg)	PEG (mg)	PEG/PCL Ratio	Length (mm)	Porosity (%)
**1**	100	56	1.8	0.0	0.0	3.7 ± 0.1	13.7 ± 3.5
**2**	100	56	1.8	0.9	0.5	4.5 ± 0.1	22.7 ± 2.1
**3**	100	56	1.8	1.8	1.0	5.8 ± 0.1	31.6 ± 2.7
**4**	100	56	1.8	3.6	2.0	8.9 ± 0.2	42.2 ± 1.6
**5**	75	42	1.8	0.0	0.0	3.7 ± 0.1	14.3 ± 2.7
**6**	75	42	1.8	0.9	0.5	4.5 ± 0.1	22.5 ± 2.0
**7**	75	42	1.8	1.8	1.0	5.7 ± 0.1	32.6 ± 2.2
**8**	75	42	1.8	3.6	2.0	9.0 ± 0.1	41.5 ± 2.5
**9**	133	56	2.4	0.0	0.0	4.8 ± 0.1	12.6 ± 1.1
**10**	133	56	2.4	1.2	0.5	6.1 ± 0.1	23.2 ± 2.3
**11**	133	56	2.4	2.4	1.0	8.1 ± 0.1	31.5 ± 2.4
**12**	133	56	2.4	4.8	2.0	11.9 ± 0.2	43.8 ± 2.8
**13**	100	42	2.4	0.0	0.0	4.7 ± 0.1	10.6 ± 2.7
**14**	100	42	2.4	1.2	0.5	6.1 ± 0.1	21.9 ± 2.0
**15**	100	42	2.4	2.4	1.0	8.1 ± 0.1	33.4 ± 1.4
**16**	100	42	2.4	4.8	2.0	11.6 ± 0.1	41.9 ± 1.8
**17**	168	56	3.0	0.0	0.0	5.8 ± 0.1	11.2 ± 1.4
**18**	168	56	3.0	1.5	0.5	7.8 ± 0.1	23.3 ± 1.2
**19**	168	56	3.0	3.0	1.0	10.3 ± 0.1	33.8 ± 0.7
**20**	168	56	3.0	6.0	2.0	14.0 ± 0.2	43.6 ± 1.6
**21**	126	42	3.0	0.0	0.0	5.8 ± 0.1	8.7 ± 0.2
**22**	126	42	3.0	1.5	0.5	7.7 ± 0.1	23.8 ± 0.9
**23**	126	42	3.0	3.0	1.0	10.2 ± 0.1	33.5 ± 0.2
**24**	126	42	3.0	6.0	2.0	14.1 ± 0.2	43.7 ± 1.0

For the PCL‐PEG/FGFb mAb@PLGA implant, PLGA was dissolved in DMSO to a final concentration of 0.1% w/v. The prepared PCL‐PEG/FGFb mAb implant was manually coated with 1 µL of the 0.1% w/v PLGA solution using a 10 µL pipette tip. The solution was evenly spread over the implant surface using gentle pipetting, and the implant was rotated during coating to ensure uniform coverage. The coated implant was then air‐dried for 1 h at room temperature.

### 4.3 Drug In Vitro Release Test

The polymeric implants were incubated in 100 µL of PBS at 37°C. At predetermined time points, the buffer was collected and replaced with fresh PBS. Antibody concentrations in the collected media were quantified using a Nanodrop spectrophotometer (Labtech, Rotherham, UK).

### 4.4 Microscopic and Scanning Electron Microscopy Evaluation

Light microscopy observations of the implants were conducted using an Olympus CKX41 inverted microscope (Olympus, Southend‐on‐Sea, UK), utilizing Olympus CellSens Standard 1.13 (Build 13479) software. For scanning electron microscopy (SEM) examination, a Quanta 200 Emission Electron Microscope (FEI Company, Hillsboro, USA) was employed. The samples were coated with a 10 nm gold layer, secured onto an aluminum stub, and visualized at an operating voltage of 10.0 kV.

### 4.5 Porosity Analysis

The porosity of the implants was measured after immersion in PBS for 7 days. The remaining amount of PEG was determined by subtracting the weight of PCL from the total remaining implant weight. The porosity was then calculated using the following Equation (1):

(1) $$Porosity\%\;=\left[1-\left(\frac{Wpcl}{\rho pcl\times Vt}+\frac{Wpeg}{\rho peg\times Vt}\right)\right]\times 100$$

*W*pcl and *W*peg are the weights of PCL and PEG in the implant, respectively; *ρ*pcl and *ρ*peg are the densities of PCL (1.145 g/cm^3^) and PEG (1.2 g/cm^3^) [69, 70], respectively; *Vt* is the total volume of the implant.

### 4.6 Machine Learning

In our machine learning input data, we included a total of 24 implant groups, with 10 replicates per group. Five distinct machine learning models were employed in this study: Random Forest (RF), Support Vector Regression (SVR), XGBoost, LightGBM, and K‐Nearest Neighbors (KNN). A five‐fold cross‐validation (5 CV) strategy was used to evaluate model performance. Each subset was used once as the validation set, while the remaining 4 subsets were used for training. Model performance was assessed using the coefficient of determination (R^2^), mean squared error (MSE), and mean absolute error (MAE). The models were executed on a computing system with the following specifications: Operating System: macOS; Processor: Apple Silicon (ARM64); Python version: 3.12.3. All models were implemented using Python within the Jupyter Notebook environment. The Scikit‐learn (version 1.6.0) library was used for training all models except for XGBoost (version 2.1.3) and LightGBM (version 4.5.0), which were trained using their respective libraries. Hierarchical clustering analysis of the Spearman correlation between selected features was performed using Ward's linkage method. The analysis was conducted with scipy (version 1.14.1), pandas (version 2.2.3), numpy (version 2.0.2), seaborn (version 0.13.2), and matplotlib (version 3.9.2). We utilized SHapley Additive exPlanations (SHAP) to interpret the contribution of individual features to model predictions. The SHAP analysis was performed using the Python SHAP package (version 0.46.0), and the SHAP values of different features were identified to assess their impact on the model's decision‐making process.

### 4.7 Cell Culture

Primary rat conjunctival fibroblasts (FFs) were cultured in an incubator at 37°C with 5% CO_2_ and 95% humidity. They were routinely passaged in T75 flasks containing complete growth media composed of DMEM X1 enriched with 10% fetal calf serum (FCS), 100 U/mL penicillin, and 0.1 mg/mL streptomycin.

### 4.8 Gel Contraction Assay

A cell suspension containing 1 × 10^5^ rat FFs was centrifuged at 1500 rpm for 5 min, and the resulting cell pellet was resuspended in 100 µL of FCS. The collagen gel mixture was formulated following a previously established technique [71]. In summary, 1 mL of Type I collagen (First Link UK Ltd, Wolverhampton, UK; 60‐30‐810) was combined with 160 µL of concentrated media composed of DMEM X10 (Merck Sigma, Dorset, UK; D2429), sodium bicarbonate 7.5% (Merck Sigma, Dorset, UK; S8761), and 2 mM L‐glutamine (Thermo Fisher Scientific, Cheshire, UK; 3102112). The pH was adjusted to 7.0 by adding 1 M sodium hydroxide (NELS, County Durham, UK; 28244–295). The resuspended fibroblasts were then incorporated into the collagen mixture, and 150 µL of the prepared solution were pipetted into each Mattek dish. The gels were left to solidify in the incubator at 37°C for 10 min. Once gel polymerization was complete, each gel was incubated with 1.4 mL of either FGFb antibody‐containing media or a control without antibody, and placed back into the incubator for further analysis. For the drug‐containing media, the optimized PCL‐PEG/FGFb mAb@PLGA implant (800 µm in diameter, 3 mm in length) was incubated in 100 µL of PBS at 37°C for specific time intervals (days 0–2, days 3–5, and days 6–30). Subsequently, two 100 µL drug release solutions, both derived from implants with identical formulations, were combined and mixed with 1.2 mL of complete media. Gel images were taken daily over the course of seven days and processed using ImageJ software. All experimental setups were performed in triplicates. The extent of matrix contraction was calculated using the following Equation (2):

(2) $$\begin{array}{ccc} & & Contraction\;Area\;\left(\%\right)\\ & & \;=\left[\frac{Initial\;gel\;area-Gel\;area\;on\;day\;n}{Initial\;gel\;area}\right]\times 100 \end{array}$$

### 4.9 RT‐qPCR

Rat FFs were plated in six‐well dishes, each well containing 1 × 10^5^ cells. FFs were incubated for 48 h in 1.4 mL of either drug‐containing media or a control without the drug. For the drug‐containing media, the optimized PCL‐PEG/FGFb mAb@PLGA implant (800 µm in diameter, 3 mm in length) was incubated in 100 µL of PBS at 37°C for specific time intervals (days 0–2, days 3–5, and days 6–30). Subsequently, two 100 µL drug release solutions, both derived from implants with identical formulations, were combined and mixed with 1.2 mL of complete media. Total RNA was isolated utilizing the QIAGEN Quick‐Start RNeasy Mini Kit (QIAGEN GmbH, Hilden, Germany; 74106), and cDNA synthesis was performed using the cDNA reverse transcription kit (Applied Biosystems, Cheshire, UK; 2754441). Quantitative real‐time PCR (RT‐qPCR) was conducted on a ViiA7 Real‐Time PCR system (Thermo Fisher Scientific, Cheshire, UK). The primers used for these reactions are detailed in Table 2. The RT‐qPCR experiment involved an initial holding phase at 50°C for 2 min and 95°C for 5 min, followed by 40 cycles at the amplification phase at 95°C for 5 s and 60°C for 30 s. The 2^−ΔΔCT^ method was used for data interpretation. Each experiment was performed in triplicates.

**TABLE 2:** Primer sequences for RT‐qPCR.

Primer	Forward	Reverse
COL1A1	ACATGTTCAGCTTTGTGGACCT	TCAGGTTTCCACGTCTCACC
COL4A1	AGAGGAGAGCCCGGTCCTAA	CTGGCCTGGAACAGCGAAAC
COL6A1	GCGGAAGAGACCATCAGCCA	AGACCCGGCTTTCCTCGTTC
FN1	TCTGGAGCCAGGAACCGAGTA	ATACCCAGGGTTGGTGACGA
TNFb2	AAATCGACATGCCGTCCCAC	CCTGGGACTGTCTGGAGCAA
ACTA2	AGCTATGTGGGGGACGAAGC	GCAAGGTCGGATGCTCCTCT
GAPDH	TCTTGTGCAGTGCCAGCCTC	TGGTAACCAGGCGTCCGATAC

### 4.10 Cell Viability

Rat FFs were plated in 96‐well plates at a concentration of 6 × 10^3^ cells per well. They were treated with a mixture of 100 µL of drug‐containing media, obtained from the optimized PCL‐PEG/FGFb mAb@PLGA implant (800 µm in diameter, 3 mm in length) on days 2, 5, and 30. After an incubation period of 24 h, the media in each well were replaced with 100 µL of fresh culture media, followed by the addition of 20 µL of CellTiter 96 AQueous One Solution Reagent (Promega, Southampton, UK; G3580) per well. The plate was then wrapped in foil and maintained at 37°C with 5% CO_2_ and 95% humidity for 2 h. Absorbance at 490 nm was recorded using a PHERAstar FS plate reader (BMG LABTECH, Aylesbury, UK), and values were normalized to the untreated control group. Each experiment was performed in triplicates.

### 4.11 Ethics Statement

All animal procedures were performed in accordance with the Association for Research in Vision and Ophthalmology (ARVO) Statement for the Use of Animals in Ophthalmic and Vision Research, and all animal experimental protocols were approved by the Home Office UK (PPL number: PP2814236).

### 4.12 Rat Model of Glaucoma Filtration Surgery

A randomised, double‐masked study was performed on 18 Sprague Dawley male rats (200–224 g, Charles River, UK). The 18 rats were randomised into 3 treatment groups (implant with FGFb antibody, implant with PBS control, MMC) using Latin square randomization. On the day of surgery, the rats were anaesthetized using an intraperitoneal injection of 75 mg/kg ketamine and 0.5 mg/kg medetomidine. Each rat was positioned on its left side, and povidone iodine 5% and proxymetacaine hydrochloride 0.5% eye drops (Bausch & Lomb, East Sussex, UK) were instilled in the right eye. A 7‐0 vicryl traction suture (Ethicon, Berkshire, UK) was placed in the upper eyelid, while the inferior lid was retracted beneath the globe to prolapse the eyeball, thereby exposing the surgical site at the superior limbus and conjunctiva. A limbal‐based conjunctival incision was made 2 mm posterior to the superior limbus, and a conjunctival flap was carefully raised using sterile vannas scissors (Surgitrac, Manchester, UK). Blunt dissection was then performed to create a conjunctival pocket. A 27G cannula (Fine Science Tools, Linton, US; 18000–10) was cut into 3‐mm segments with one end bevelled, and a 10‐0 Ethilon nylon suture (Ethicon, Berkshire, UK) was placed through the tube at two‐thirds from the bevelled end without occluding the lumen. The suture was then passed through the partial‐thickness sclera to secure the tube. The anterior chamber was entered using a 26G needle (BD, Berkshire, UK), and aqueous humor egress was confirmed upon needle withdrawal. The bevelled end of the tube was inserted through the needle track to establish a fistula between the anterior chamber and subconjunctival space, and the tube was sutured to the sclera using the pre‐fixed 10‐0 Nylon suture. Tenon's capsule was gently wrapped over the tube, and a sterilized implant, either with FGFb antibody or PBS control, was placed in the conjunctival pocket. In the MMC group, a surgical sponge soaked with MMC (0.2 mg/ml) (Fisher Scientific, Leicestershire, UK; BP2531‐2) was applied to the exposed sclera for 2 min. After sponge removal, irrigation of the ocular surface and subconjunctival space was performed using 20 mL of physiologic balanced salt solution (Alcon Laboratories, Fort Worth, TX, USA) [2, 72]. The conjunctiva was then closed with two interrupted 10‐0 nylon sutures, and a filtration bleb was observed immediately post‐surgery [73]. Postoperative care included the application of chloramphenicol 1% ointment to the right operated eye, and subcutaneous injections of 5 mg/kg carprofen for analgesia and atipamezole 1 mg/kg for reversal of the general anaesthesia. The rats were examined every 3 days by two double‐masked blinded researchers. At each postoperative visit (days 3, 6, 9, 12, 14), intraocular pressure (IOP), bleb survival, bleb vascularity (0: avascular; 1: normal; 2: hyperaemic; 3: very hyperaemic), corneal and conjunctival toxicity, and anterior chamber inflammation were assessed. On day 14, the rats were sacrificed, and both eyes were enucleated for further analysis.

### 4.13 Histology

The eyeballs were fixed in 10 % buffered formalin (CellPath, Powys, UK; 03830858) for 24 h and preserved in 70% ethanol until they were embedded in paraffin for histological analysis. Paraffin sections of 4 µm thickness were cut from the bleb tissue at 0.75 mm from the glaucoma tube. The tissue sections underwent hematoxylin and eosin, Gomori's trichrome, picrosirius red, and alpha‐smooth muscle actin (αSMA) staining [2, 74]. Tissue block preparation and histological staining were conducted at the NIHR Great Ormond Street Hospital Biomedical Research Centre (London, UK). All the right operated eyes were compared to the left non‐operated eyes, which were used as controls for the appearance of normal conjunctival tissue. The sections were graded using a modified grading system from −4 to +4 (0: same as control eye; 1: 1–25% difference from control eye; 2: 26–50% difference from control eye; 3: 51–75% difference from control eye; 4: 76–100% difference from control eye; prefix +, more than; prefix ‐, less than) [75].

### 4.14 Statistical Analysis

Statistical analyses were performed using GraphPad Prism 10. The level of significance was set as *p* < 0.05. One‐way ANOVA with Tukey's post hoc test was used for comparing multiple groups, and Student's unpaired *t*‐test was used for comparing two groups. Data were shown as mean ± SD (N ≥ 3).

## Author Contributions

Mengqi Qin: Writing – original draft, formal analysis, investigation, methodology, software, conceptualization. Wenbing Jiang: Writing – review and editing, formal analysis, investigation. Kai Thong: Writing – review and editing, methodology, data curation. Zeynep Ulker: Writing – review and editing, software, data curation, methodology. Brihitejas Patel: Writing – review and editing, methodology. Cynthia Yu‐Wai‐Man: Writing – review and editing, supervision, resources, funding acquisition, methodology, conceptualization.

## Funding

This work is funded by the Medical Research Council (grant numbers MR/T027932/1 and MR/Z504841/1).

## Conflicts of Interest

The authors declare no conflicts of interest.

## Supporting information




**Supporting File**: adhm70754‐sup‐0001‐SuppMat.docx.

## Data Availability

The data that support the findings of this study are available from the corresponding author upon reasonable request.
