# Supplementary material for: Precise Control of Drug Release in Machine Learning‐Designed Antibody‐Eluting Implants for Postoperative Scarring Inhibition in Glaucoma
Source: Adv Healthc Mater. 2026 Jan 21;15(13):e02689. doi: 10.1002/adhm.202502689 (PMC13058792; doi:10.1002/adhm.202502689)
Supplement: Supplementary file 1 — Supporting File: adhm70754‐sup‐0001‐SuppMat.docx. [file ADHM-15-0-s001.docx]

Supporting Information

Title

Precise Control of Drug Release in Machine Learning-Designed Antibody-Eluting Implants for Postoperative Scarring Inhibition in Glaucoma

### Mengqi Qin^1^, Wenbing Jiang^1^, Kai Xin Thong^1^, Zeynep Ulker^1^, Brihitejas Patel^1^, Cynthia Yu-Wai-Man^1^

1. Faculty of Life Sciences & Medicine, King’s College London, London, SE1 1UL, UK.

**Corresponding Author:**

Dr Cynthia Yu-Wai-Man, MBBS FRCOphth PhD

King’s College London

London SE1 1UL, UK

Telephone: +44 (0)20 7848 6666

[cynthia.yu-wai-man@kcl.ac.uk](mailto:cynthia.yu-wai-man@kcl.ac.uk)

**Methods**

***Detection of the IgG particle size***

Test formulations were dispersed in 1 mL of deionized water to the final concentration of 10 μg/mL, and the resulting suspension was transferred into a disposable folded capillary cell (Malvern Panalytical Ltd, Malvern, UK; DTS1070). Particle sizes were determined using dynamic light scattering (Malvern Panalytical Ltd, Malvern, UK; Zetasizer Pro). Measurements were performed in triplicates for each sample.

***Gel electrophoresis***

Twenty μL of the drug releasing solution (1 μg/μL) or pure IgG (1 μg/μL) were mixed with 10 μL of non-reducing SDS (Thermo Fisher Scientific, Cheshire, UK; J63615.AD), 4 μL of DTT (Thermo Fisher Scientific, Cheshire, UK; R0861), and 6 μL of PBS. The prepared mixtures were heated at 80 °C for 5 minutes in a thermoblock. Afterwards, 40 μL from each sample were loaded into individual wells of a Bis-Tris Plus gel (4–12%) (Thermo Fisher Scientific, Cheshire, UK; NW0412) for electrophoresis, which was run at 150 V for 45 minutes. Subsequently, the gel was immersed in a plastic container containing Briliant blue R staining solution (Merck Sigma, Dorset, UK; B6529) and left for 2 hours. The gel was washed out with acetic acid glacial (VWR International, Leicestershire, UK; 64-19-7) overnight before being imaged.

***Gene Expression Analysis and Protein-Protein Interaction (PPI) Network***

Gene expression levels of *FGFb* and *TGF-β2* were analyzed in fibroblasts from different regions of the aqueous shunt implantation site, including scleral fibroblasts (hSF), choroidal fibroblasts (hCF), and Tenon’s space fibroblasts (hTF), using publicly available data provided by Löbler et al. (GSE40929) ^[53]^. Additionally, the expression levels of *FGFb* and *TGF-β2* were assessed in conjunctival tissues, primary conjunctival epithelial cells (PCEC), and two conjunctival epithelial cell lines (IOBA-NHC and ChWK), using data provided by Tong et al. (GSE8633) ^[54]^. A protein-protein interaction network for FGFb was constructed using the STRING database (https://string-db.org/), and the network was visualized and analyzed using Cytoscape (version 3.10.2).


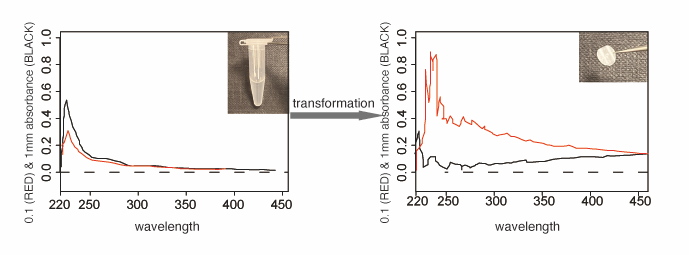


**Figure S1:** UV-Vis spectra of the polymer-drug mixture were measured before (liquid state) and after (solid state) DCM evaporation.


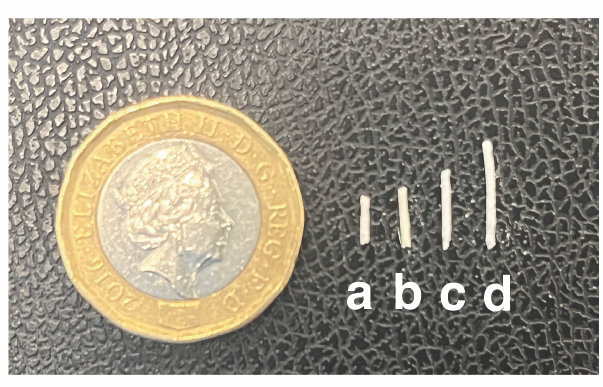


**Figure S2:** Representative images of fabricated implants with varying PCL and PEG/PCL ratios: (a) PCL = 2.4 mg, PEG/PCL = 0; (b) PCL = 2.4 mg, PEG/PCL = 0.5; (c) PCL = 2.4 mg, PEG/PCL = 1; (d) PCL = 2.4 mg, PEG/PCL = 2.

**
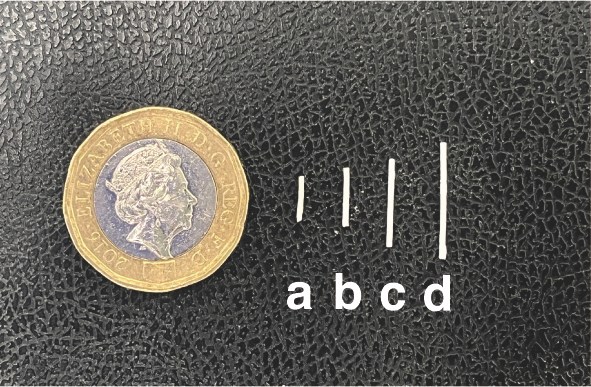
**

**Figure S3:** Representative images of fabricated implants with varying PCL and PEG/PCL ratios: (a) PCL = 3.6 mg, PEG/PCL = 0; (b) PCL = 3.6 mg, PEG/PCL = 0.5; (c) PCL = 3.6 mg, PEG/PCL = 1; (d) PCL = 3.6 mg, PEG/PCL = 2.


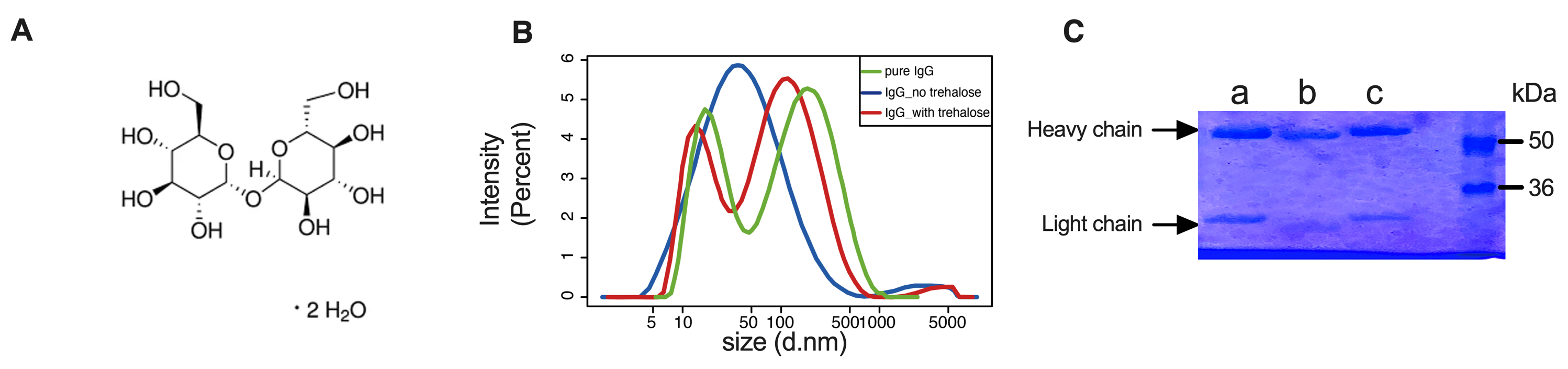


**Figure S4:** The protective effect of trehalose (10% w/v) on IgG and IgG released from the implant with different compositions. (A) Chemical structure of trehalose. (B) IgG size distribution measured by Zetasizer. Green line: pure IgG. Blue line: IgG released from the implant without trehalose protection. Red line: IgG released from the implant with trehalose protection. (C) SDS-PAGE after staining with Coomassie Blue comparing pure IgG and IgG released from implants with or without trehalose protection, a: pure IgG; b: IgG released from the implant without trehalose protection; c: IgG released from the implant with trehalose protection.


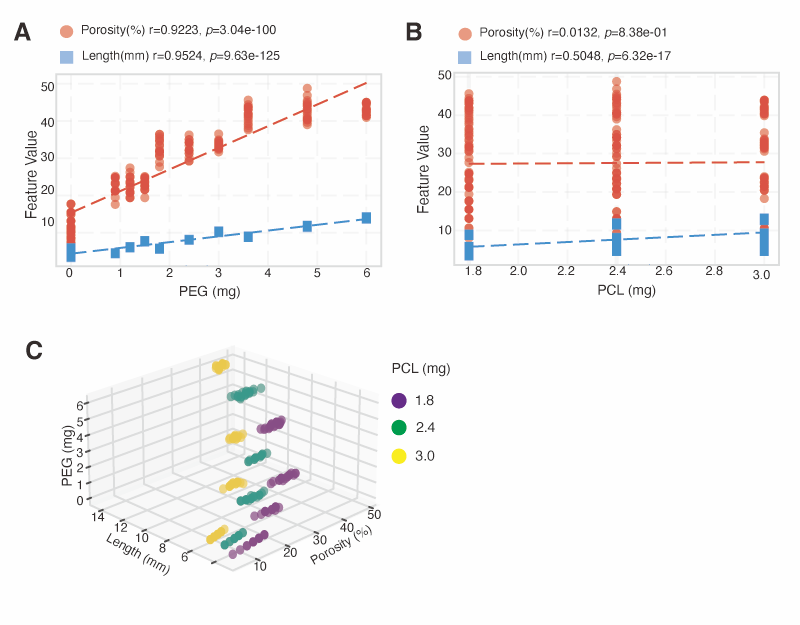


**Figure S5:** Relationship between implant length, porosity, PEG content and PCL content. (A) Scatter plot of PEG content against implant length (mm) (blue) and porosity (%) (red), with a fitted regression line. (B) Scatter plot of PCL content against implant length (mm) (blue) and porosity (%) (red), with a fitted regression line. (C) Scatter plot with marginal histograms illustrating the distribution of key implant features: PEG content (mg), PCL content (mg), porosity (%), and implant length (mm).

**
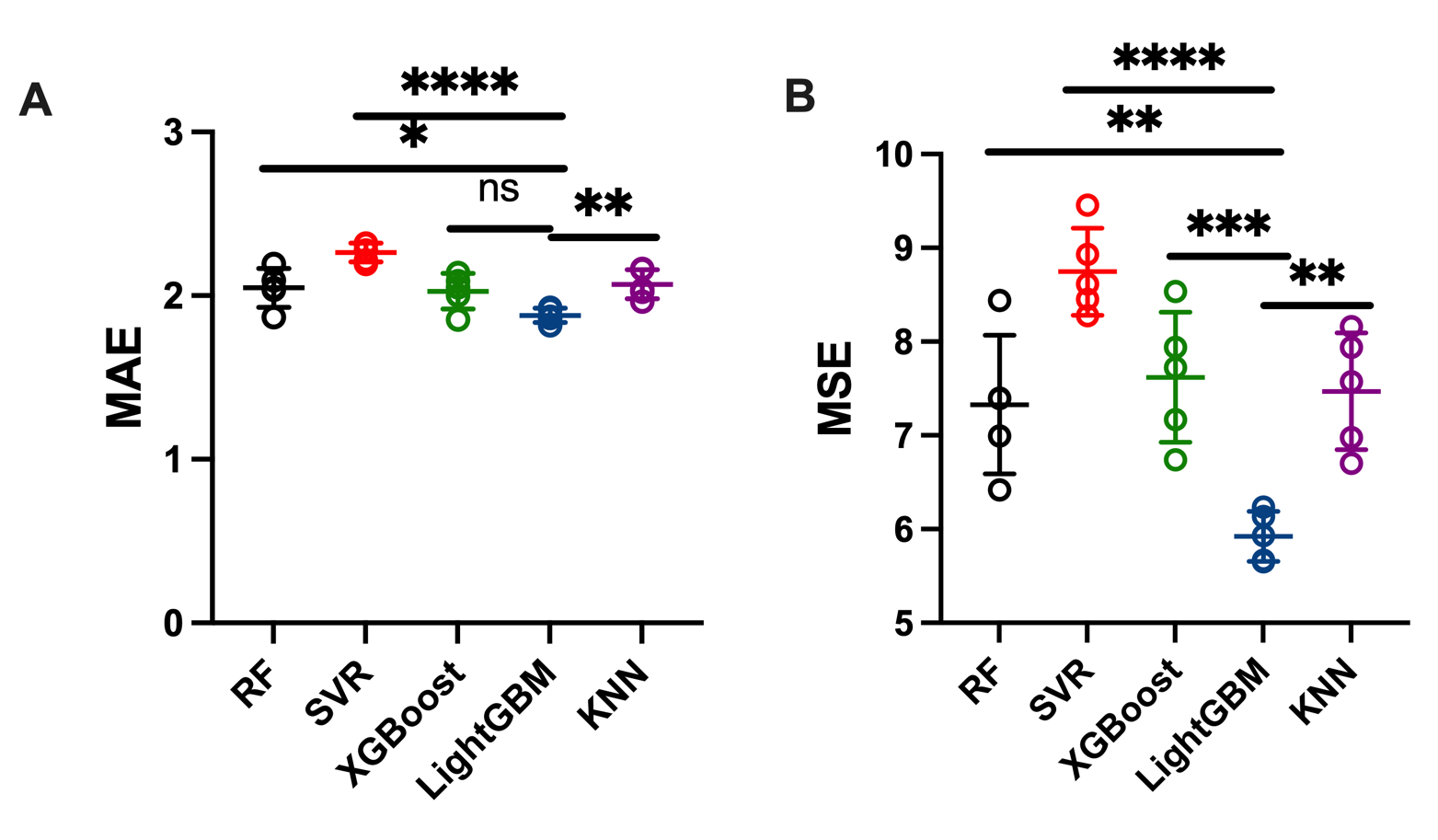
**

**Figure S6:** Assessment of different models (RF, SVR, XGBoost, LightGBM, KNN) for predicting drug release based on input length, IgG loading (μg), PEG content (mg), PCL content (mg), and release time. (A) MSE (Mean Squared Error) scores for predicted drug release profiles generated by different models. (B) MAE (Mean Absolute Error) scores for predicted drug release profiles generated by different models.


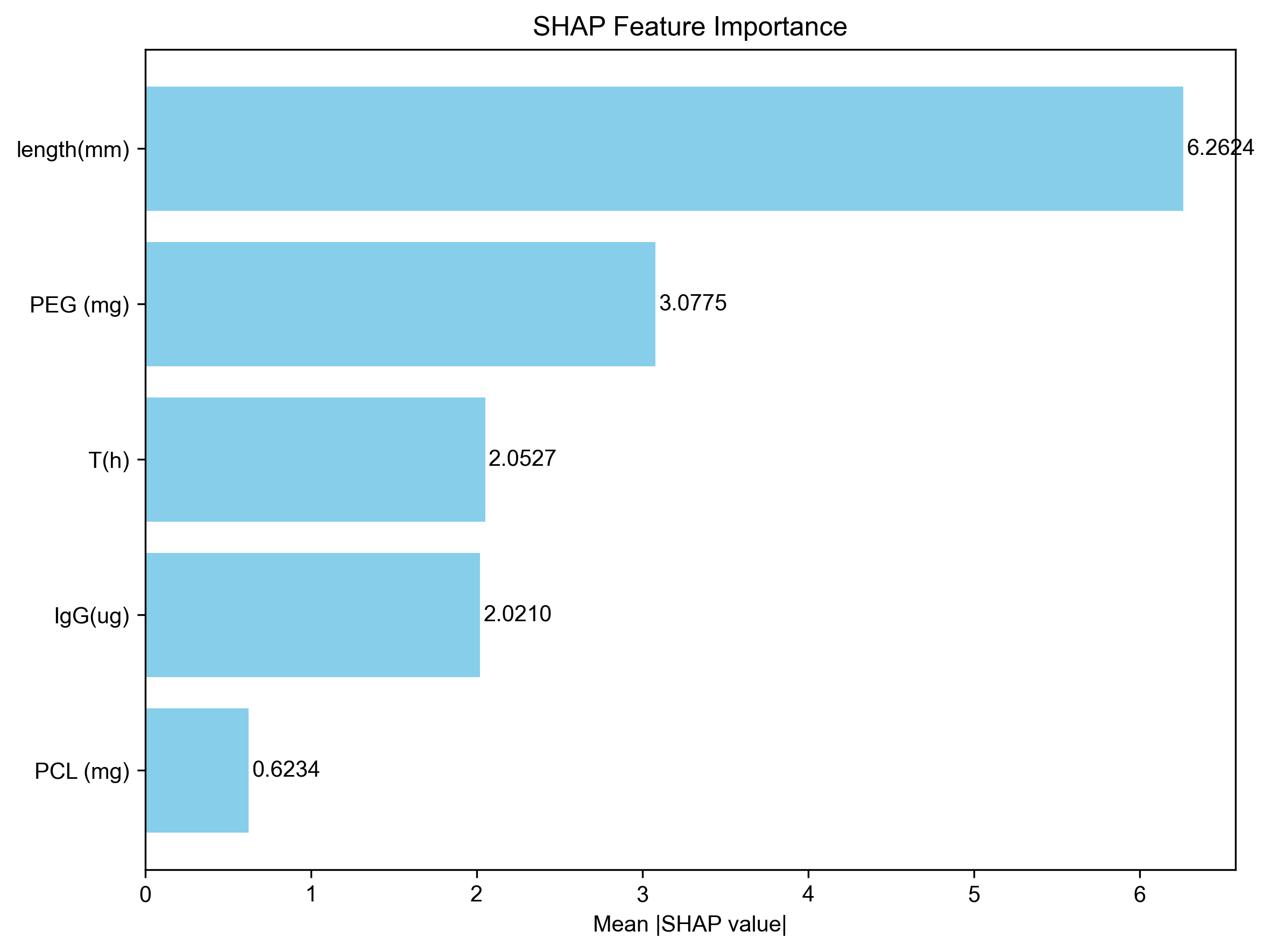


**Figure S7:** SHAP bar plot for LightGBM showing the contribution of different features to drug release.


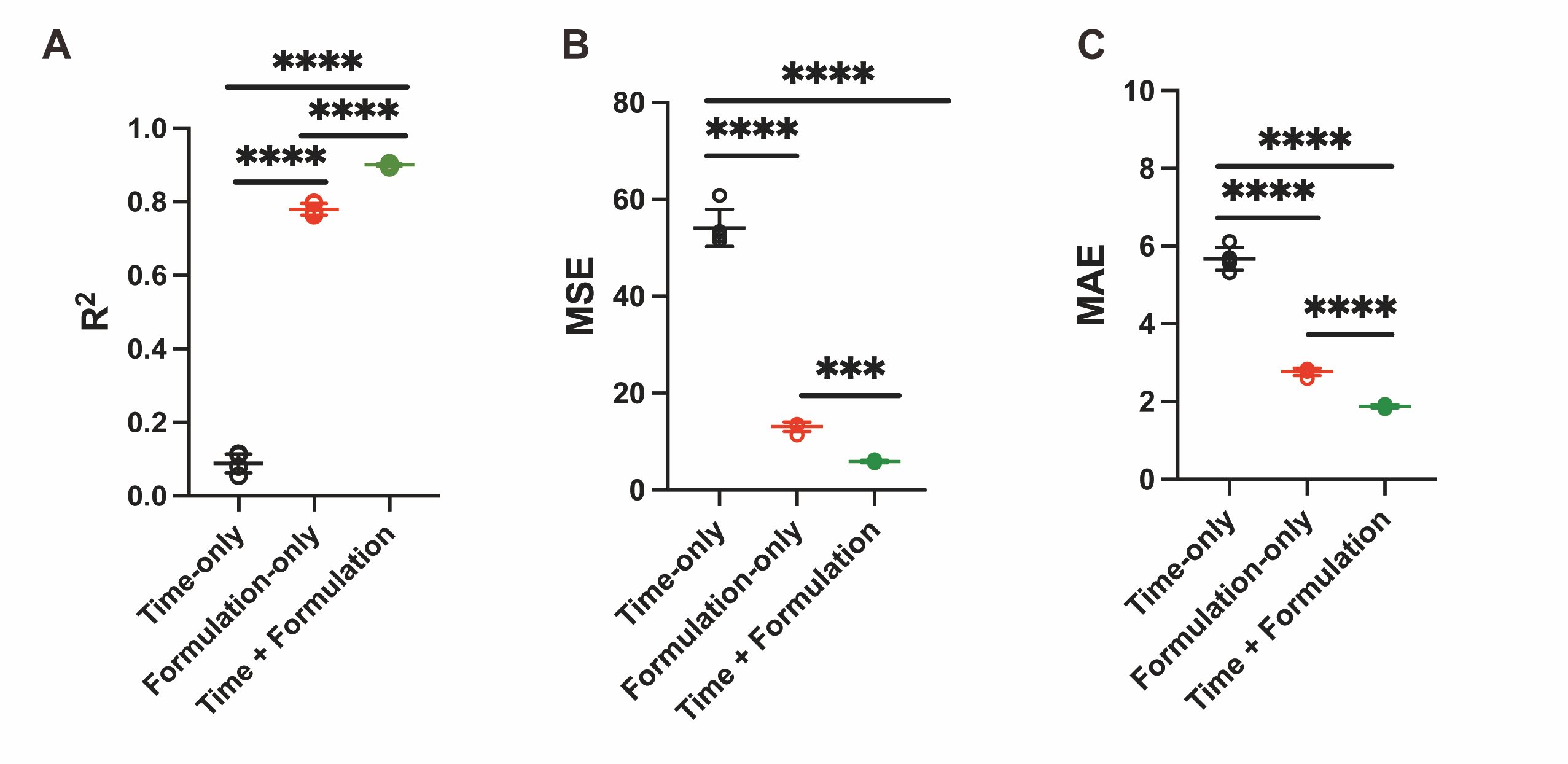


**Figure S8.** Feature ablation analysis comparing the predictive performance of LightGBM models trained with different input feature sets. (A) R², (B) MSE, and (C) MAE of models trained using release time alone, formulation parameters (length, PEG, PCL, IgG) alone, and the combined feature set of release time and formulation parameters. ****p* < 0.001, *****p* < 0.0001. One-way ANOVA with Tukey post hoc test is shown.

**
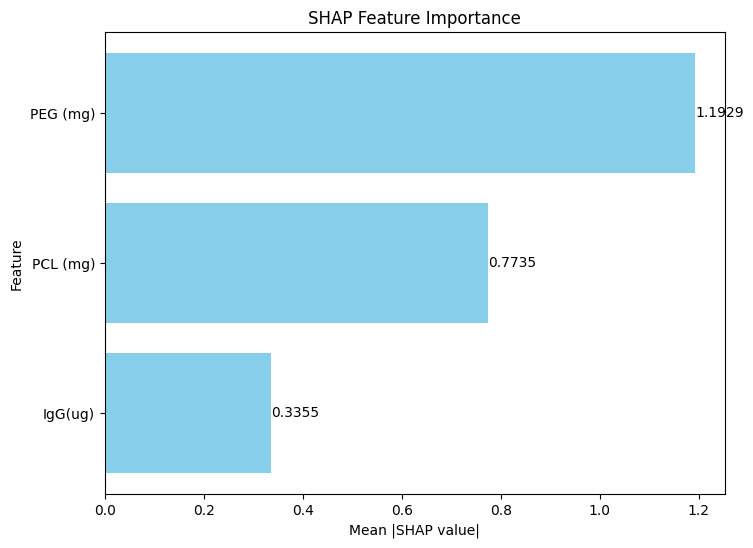
**

**Figure S9:** SHAP bar plot for LightGBM showing the contribution of different features to the cumulative drug release per millimeter of implant.


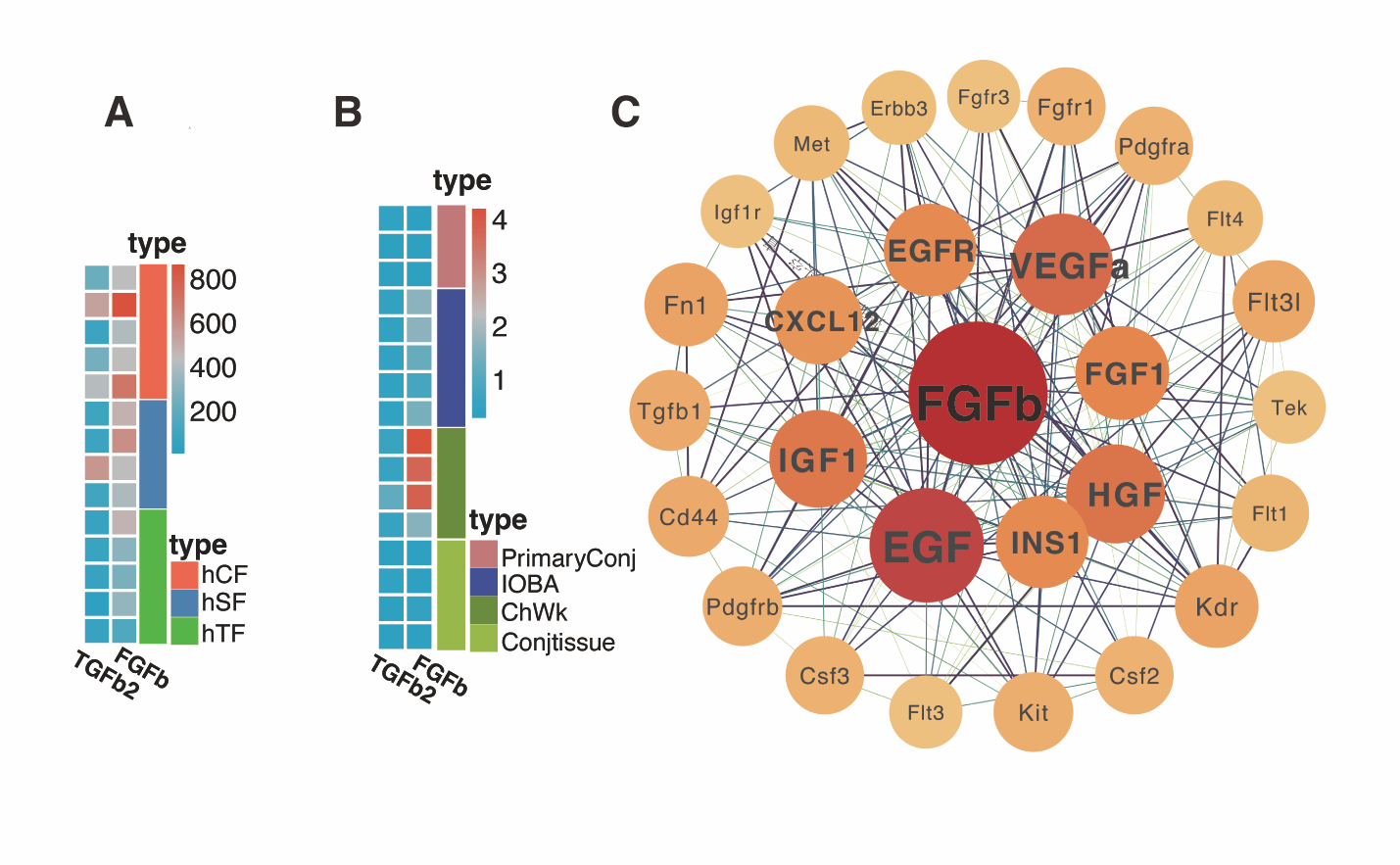


**Figure S10:** FGFb expression in conjunctival tissues and its biological activity. (A) Gene expression levels of *FGFb* and *TGF-β2* in fibroblasts from various regions of the aqueous shunt implantation site, including scleral fibroblasts (hSF), choroidal fibroblasts (hCF), and Tenon’s space fibroblasts (hTF) (B) *FGFb* and *TGF-β2* expression levels in conjunctival tissues, primary conjunctival epithelial cells (PCEC), and two conjunctival epithelial cell lines (IOBA-NHC and ChWK) (C) Protein-protein interaction (PPI) network analysis for FGFb. Nodes represent proteins and edges represent interactions between proteins. The size and color of each node correspond to its degree value, which indicates the number of direct interactions a protein has within the network. Larger and redder nodes represent proteins with higher degree values (more interactions), while smaller and yellower nodes represent proteins with lower degree values (fewer interactions).

**Table S1:** MSE (Mean Squared Error) scores for predicted drug release profiles generated by different models (RF, SVR, XGBoost, LightGBM, KNN). Input: PEG content (mg), PCL content (mg), IgG loading (μg), IgG/PCL ratio; Output: cumulative drug release (per millimeter of implant).

| **MSE** | | | | | |
| --- | --- | --- | --- | --- | --- |
|  | **RF** | **SVR** | **XGBoost** | **LightGBM** | **KNN** |
| **Fold 1** | 0.4719 | 0.4998 | 0.4620 | 0.4104 | 0.4311 |
| **Fold 2** | 0.3338 | 0.3771 | 0.3372 | 0.3479 | 0.3243 |
| **Fold 3** | 0.1885 | 0.2935 | 0.1905 | 0.2006 | 0.2075 |
| **Fold 4** | 0.3705 | 0.2533 | 0.3671 | 0.3287 | 0.4181 |
| **Fold 5** | 0.2678 | 0.3120 | 0.2624 | 0.2797 | 0.2756 |
| **Mean ± SD** | 0.3265  ±0.1049 | 0.3471  ±0.0916 | 0.3238  ±0.1033 | 0.3135  ±0.0797 | 0.3313  ±0.0918 |

**Table S2:** MAE (Mean Absolute Error) scores for predicted drug release profiles generated by different models (RF, SVR, XGBoost, LightGBM, KNN). Input: PEG content (mg), PCL content (mg), IgG loading (μg), IgG/PCL ratio; Output: cumulative drug release (per millimeter of implant).

| **MAE** | | | | | |
| --- | --- | --- | --- | --- | --- |
|  | **RF** | **SVR** | **XGBoost** | **LightGBM** | **KNN** |
| **Fold 1** | 0.4106 | 0.4213 | 0.4059 | 0.4082 | 0.4049 |
| **Fold 2** | 0.4002 | 0.4658 | 0.4021 | 0.4144 | 0.3945 |
| **Fold 3** | 0.3309 | 0.3885 | 0.3350 | 0.3505 | 0.3412 |
| **Fold 4** | 0.4136 | 0.3706 | 0.4120 | 0.3877 | 0.4388 |
| **Fold 5** | 0.3466 | 0.3976 | 0.3443 | 0.3591 | 0.3495 |
| **Mean**  **± SD** | 0.3804  ±0.0381 | 0.4088  ±0.0368 | 0.3799  ±0.0358 | 0.3840  ±0.0286 | 0.3858  ±0.0383 |
